# Comparative Genomic Characterization of *Francisella tularensis* Strains Belonging to Low and High Virulence Subspecies

**DOI:** 10.1371/journal.ppat.1000459

**Published:** 2009-05-29

**Authors:** Mia D. Champion, Qiandong Zeng, Eli B. Nix, Francis E. Nano, Paul Keim, Chinnappa D. Kodira, Mark Borowsky, Sarah Young, Michael Koehrsen, Reinhard Engels, Matthew Pearson, Clint Howarth, Lisa Larson, Jared White, Lucia Alvarado, Mats Forsman, Scott W. Bearden, Anders Sjöstedt, Richard Titball, Stephen L. Michell, Bruce Birren, James Galagan

**Affiliations:** 1 Microbial Analysis Group, Broad Institute of MIT and Harvard, Cambridge, Massachusetts, United States of America; 2 Department of Biochemistry and Microbiology, University of Victoria, Victoria, British Columbia, Canada; 3 Center for Microbial Genetics and Genomics, Northern Arizona University, Flagstaff, Arizona, United States of America; 4 Pathogen Genomics Division, Translational Genomics Research Institute, Phoenix, Arizona, United States of America; 5 Department of CBRN Defense and Security, Swedish Defense Research Agency, Umeå, Sweden; 6 Centers for Disease Control and Prevention, Division of Vector-Borne Infectious Diseases, Fort Collins, Colorado, United States of America; 7 Department of Clinical Bacteriology, Clinical Microbiology, Umeå University, Umeå, Sweden; 8 University of Exeter, School of Biosciences, Exeter, United Kingdom; 9 Boston University Department of Biomedical Engineering and the Boston University Medical School, Boston, Massachusetts, United States of America; University of Toronto, Canada

## Abstract

Tularemia is a geographically widespread, severely debilitating, and occasionally lethal disease in humans. It is caused by infection by a gram-negative bacterium, *Francisella tularensis*. In order to better understand its potency as an etiological agent as well as its potential as a biological weapon, we have completed draft assemblies and report the first complete genomic characterization of five strains belonging to the following different *Francisella* subspecies (subsp.): the *F. tularensis* subsp. *tularensis* FSC033, *F. tularensis* subsp. *holarctica* FSC257 and FSC022, and *F. tularensis* subsp. *novicida* GA99-3548 and GA99-3549 strains. Here, we report the sequencing of these strains and comparative genomic analysis with recently available public *Francisella* sequences, including the rare *F. tularensis* subsp. *mediasiatica* FSC147 strain isolate from the Central Asian Region. We report evidence for the occurrence of large-scale rearrangement events in strains of the *holarctica* subspecies, supporting previous proposals that further phylogenetic subdivisions of the Type B clade are likely. We also find a significant enrichment of disrupted or absent ORFs proximal to predicted breakpoints in the FSC022 strain, including a genetic component of the Type I restriction-modification defense system. Many of the pseudogenes identified are also disrupted in the closely related rarely human pathogenic *F. tularensis* subsp. *mediasiatica* FSC147 strain, including modulator of drug activity B (*mdaB*) (FTT0961), which encodes a known NADPH quinone reductase involved in oxidative stress resistance. We have also identified genes exhibiting sequence similarity to effectors of the Type III (T3SS) and components of the Type IV secretion systems (T4SS). One of the genes, *msrA2* (FTT1797c), is disrupted in *F. tularensis* subsp. *mediasiatica* and has recently been shown to mediate bacterial pathogen survival in host organisms. Our findings suggest that in addition to the duplication of the *Francisella* Pathogenicity Island, and acquisition of individual loci, adaptation by gene loss in the more recently emerged *tularensis*, *holarctica*, and *mediasiatica* subspecies occurred and was distinct from evolutionary events that differentiated these subspecies, and the *novicida* subspecies, from a common ancestor. Our findings are applicable to future studies focused on variations in *Francisella* subspecies pathogenesis, and of broader interest to studies of genomic pathoadaptation in bacteria.

## Author Summary

Tularemia is a zoonotic disease that is widely disseminated throughout the Northern Hemisphere and is caused by different strain types of bacteria belonging to the genus Francisella. In general, *Francisella tularensis* subspecies are able to infect a wide range of mammals including humans and are often transmitted via insect vectors such as ticks. Depending on the strain and route of infection the disease may be fatal in humans. In order to better understand *F. tularensis* as an etiological agent as well as its potential as a biological weapon, we have completed draft sequence assemblies of five globally diverse strains. We have performed a comparative analysis of these sequences with other available public *Francisella* sequences of strains of differing virulence. Our analysis suggests that genome rearrangements and gene loss in specific *Francisella* subspecies may underlie the evolution of niche adaptation and virulence of this pathogen.

## Introduction

*Francisella tularensis* is a Gram-negative, facultative intracellular bacterium and its ability to survive and grow within macrophages is a trait that contributes to its virulence. Virulent isolates of the bacterium are the etiological cause of tularemia, a severely debilitating and occasionally fatal disease in humans. Transmission can occur by aerosolization when infected animal carcasses are disrupted, entry through skin abrasions or sites of bites from an arthropod vector, or by ingestion of contaminated food or water. As few as 10 cells have been found to cause respiratory tularemia, making *F. tularensis* one of the most infectious pathogens known at present [1],[2]. The effective dose of infection has contributed to past efforts to develop bioweapons containing the *F. tularensis* bacterium, and due to the particularly high mortality rate of respiratory tularaemia, there is still concern that weapons of this nature still exist [3].

Genetic and spatial diversity patterns among a variety of *Francisella* strain isolates have been previously reported and together with phylogenetic analyses, have provided much insight into the evolutionary divergence of the Francisella genus [4]–[6]. Francisella is the only genus of the family *Francisellaceae*, and has no close pathogenic relatives [7]. The divergent nature of the *F. tularensis* lineage is evident from phylogenetic studies examining a subset of homologous genes and proteins present in *Francisella* and 15 other genomes from species also belonging to the γ subclass of proteobacteria [8],[9]. The variation of previously characterized genetic attributes between different *F. tularensis* subspecies (subsp.) is generally minor, despite the more distinct variations in virulence and geographical origin. Previous phylogenetic studies have examined the relationships between the subspecies of *Francisella* and have recently demonstrated that there are distinct clades of the *F. tularensis* subsp. *tularensis* (Type A) lineage, Type A.I and Type A.II [4],[10],[11]. Divergence of the Type A strains predated the *F. tularensis* subsp. *holarctica* FSC022 *japonica* strain, which is distinct from the main *F. tularensis* subsp. *holarctica* (Type B) radiation lineage [5],[6],[12].

Studies of strain dispersion and divergence have provided insight into likely migration histories of different *Francisella* lineages. It has been proposed that the A.I strains originated in the Midwestern North American region prior to the emergence of the A.II strains [11]. The subsequent divergence of the *F. tularensis* subsp. *holarctica* biovar *japonica* strain likely occurred prior to the other Type B strains (reviewed in [12]). Although *F. tularensis* subsp. *novicida* has been isolated in Thailand and Australia, the geographical distribution of *F. tularensis* generally spans the Northern Hemisphere and the most virulent subspecies, *F. tularensis* subsp. *tularensis* (Type A) is found exclusively in North America. Cluster analysis of microarray hybridization data has shown overall genomic similarities between *F. tularensis* subsp. *tularensis* and *F. tularensis* subsp. *mediasiatica* strains, even though strains of the latter subspecies are geographically distinct and are distinguishable by their moderate virulence for mammals [13]. *F. tularensis* subsp. *mediasiatica* strains have only been isolated from Kazakhstan and Turkmenistan in Central Asia. This subspecies is virulent in mice [14] and is thought to be more closely related to the highly virulent *Francisella tularensis* subspecies *tularensis*. However, *F. tularensis* subsp. *mediasiatica* is believed to be of relatively low virulence in humans, and only rare cases of human disease caused by this subspecies are known. *F. tularensis* subsp. *mediasiatica* virulence, therefore, more closely resembles that of *F. tularensis* subsp. *holarctica* strains (reviewed in [12]). The subspecies *F. tularensis* subsp. *holarctica* (Type B) is generally more benign than *F. tularensis* subsp. *tularensis* (Type A) strains and has been used to develop the potential vaccine strain, LVS [11],[12],[15],[16].

Strain divergence in *Francisella* subspecies is likely due to smaller scale genetic differences including those previously characterized in pathogenicity gene clusters and individual gene families, although the biological significance of these variations in regulating virulence remains to be deciphered [9],[13],[17],[18]. It has, however, been well established from evolutionary studies of bacterial pathogens that both gene gain and loss can contribute to virulence as well as pathoadaptation to specific hosts [19]. Virulence and host-range can be influenced by the acquisition of genes that are either structurally organized into pathogenicity islands, or distributed throughout in the genome [20]. And the acquisition of virulence gene clusters often marks the evolutionary differentiation of bacterial pathogens from nonpathogenic ancestors (reviewed in [19]). A duplication of a cluster of genes characterized in *Francisella* as the *Francisella* Pathogenicity Island (FPI), for example, could have contributed to the differentiation of the more pathogenic *Francisella* Type A and B strains from the human non-pathogenic, or rarely pathogenic *F. tularensis* subsp. *novicida* and *F. philomiragia* strains [18].

Although pathoadaptation by gene loss is usually thought of as an opposing evolutionary force to gene acquisition, the presence of both mechanisms may be advantageous for dynamic host niche colonization by bacteria. There is evidence that loss of gene function, as evident by a higher abundance of pseudogenes in the genome, can promote either increased virulence or attenuation [19],[21]. An evolutionary fluctuation of niche adaptation is likely due to a lack of selective pressures for genes encoding functions specialized to certain host environments [22]–[24]. In the case of *Francisella*, previous studies have suggested that the genomes of more recently diverged subspecies may have adapted to intramacrophage growth via disruptions in many genes (pseudogenes) that include protein products involved in DNA metabolism, amino acid biosynthesis and transport [17],[25]. The increased presence of pseudogenes has also been correlated with a high frequency of Insertion Sequence (IS) elements and genome rearrangements in more virulent strains of numerous bacterial pathogens, including strains of *Francisella* [17]. Specifically, comparison of the more ancestral *F. tularensis* subsp. *novicida* U112 strain with *F. tularensis* subsp. *tularensis* Schu S4 and the *F. tularensis* subsp. *holarctica* LVS identified multiple IS elements associated genomic rearrangements and a collection of genes specific to the human pathogenic strains [17]. Extensive rearrangements have been characterized in *Francisella* and are known to have occurred from analysis across different subspecies (e.g., OSU18 vs SCHU S4) [26], as well as from comparisons across different clades of the Type A subspecies (WY96-3418 vs SCHU S4) [10]; reviewed in [25]. However, to date, there has been no reported evidence for rearrangements from comparisons among whole genome sequences of the Type B lineage.

Previous examination of gene acquisition and loss occurring prior to the divergence of the human pathogenic *tularensis* (Schu S4) and *holarctica* (LVS) strains by comparison to a nonpathogenic *novicida* relative (U112), identified numerous factors potentially involved in human infection (16). Since these strains are quite phylogenetically distant from one another and are all isolates from *Francisella* subspecies-rich geographical locales, this comparison is of limited value in identifying potential factors required for lethal human infection by the Type A subtype strains. Now, the recently available sequences of the geographically distinct and moderately human pathogenic FSC022 Type B strain from Japan as well as the closely related rarely human pathogenic *F. tularensis* subsp. *mediasiatica* FSC147 strain from the Central Asian Region has enabled a more comprehensive comparison between highly virulent, human pathogenic and human-non-(or rarely) pathogenic strains. Our studies, therefore, provide new insights into how structural genomic rearrangements has contributed to the acquisition and loss of factors regulating virulence and pathoadaptation of different *Francisella* subspecies, and how shared polymorphisms between the more recently emerged *mediasiatica* and *holarctica* subspecies might be signatures of attenuation.

## Results/Discussion

### General Features of Five Sequenced *Francisella* Genomes

We have gleaned further insight into subspecies specific differences in gene content with a comprehensive comparative analysis of 20 *Francisella* strains. Included in our analysis are new genome sequences for five *Francisella tularensis* strains.

**Figure 1:**
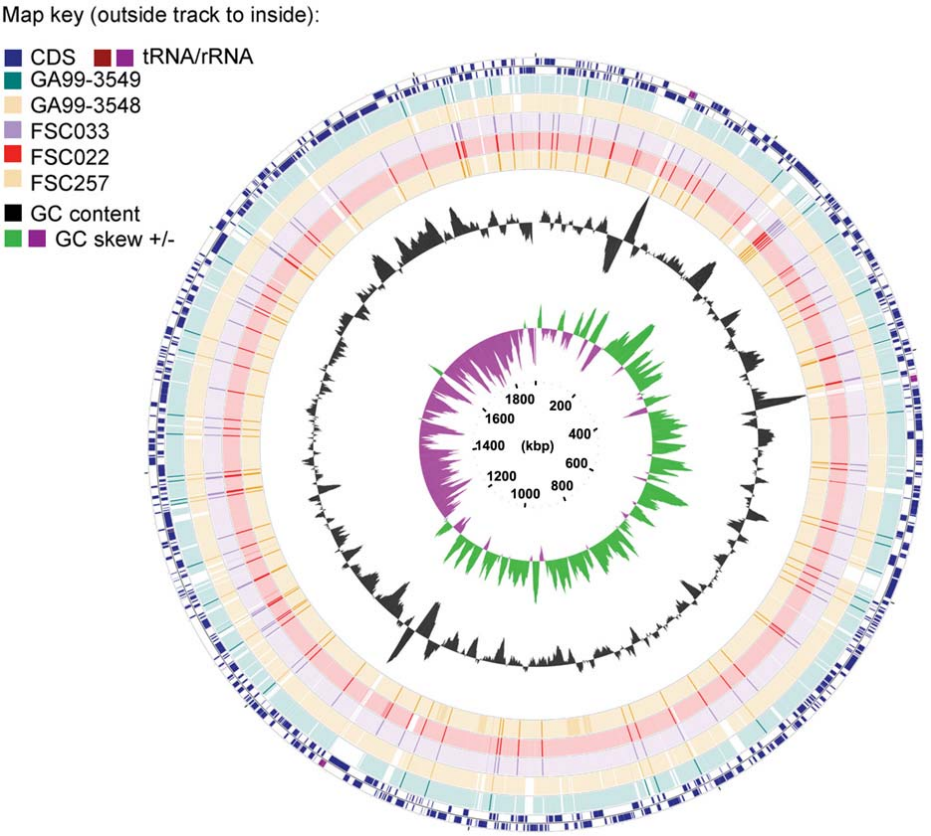
Pairwise alignments between five new *Francisella* genome sequences and a reference genome exhibit >95% sequence conservation. Genome comparative maps were constructed using CGview software to map pairwise blastn alignments between several *Francisella* genomes (minimum percent identity = 95 and expected threshold = 1e-5). Specifically, five newly sequenced Francisella genomes (*F. tularensis* subsp. *holarctica* FSC257 and FSC022; *F. tularensis* subsp. *tularensis* FSC033; and *F. tularensis* subsp. *novicida* GA99-3548, and GA99-3549 strains) were aligned to the *F. tularensis* subsp. *holarctica* OSU18 reference sequence (outside blue track of genome map). A high degree of similarity between the genomes (>95%) is evident from the continuous blocks of synteny (colored regions).

The draft genomic sequences of *F. tularemia* subsp. *tularensis* strain FSC033, *F. tularemia* subsp. *holarctica* strains: FSC022, and FSC257, and *F. tularemia* subsp. *novicida* strains: GA99-3548 and GA99-3549 have been annotated and deposited in GenBank (Materials and Methods). Genome and assembly statistics for each strain are summarized in Table 1. All of the genomes consist of a single circular chromosome and are approximately 2 Mb in size. Although the five strains represent different subspecies of *Francisella*, the overall features of the genomes are quite similar (Table 1). The average GC content and distribution is consistent with previous studies reporting the lower G+C content in *Francisella* [9]. The average number of genes is 1,730, with a mean of 1,574 total protein-coding genes. The genomes of the *novicida* subspecies carry the highest percentage of intact ORFs (97%) and conversely, the *F. tularensis* subsp. *holarctica* FSC257 strain sequence carries the lowest percentage (84%) in comparison to the other five genomes. Pairwise alignment using blastn (1e^-5^, 95%) of the draft genomes with the *F.tularemia* subsp. *holarctica* OSU18 reference genome shows the high level of overall similarity between genomes across subspecies (>95%) (Figure 1). The average gene length does vary across the different strains of *Francisella* and is correlated with the abundance of pseudogenes (Table 1). We also report a significantly larger number of total transposable elements present in the Type A and Type B strains in comparison to the *novicida* strains, which is consistent with previous studies [17]. It is worth noting that the highest numbers are present in Type B strains, even though rearrangement events have not been characterized by previous comparisons between these genomes.

**Table 1:** Gene and Assembly Statistics Summary.

Human virulence	low	low	high	rarely	rarely
Strains	F. *tularensis* subsp. *holarctica* 257	F. *tularensis* subsp. *holarctica* FSC022	F. *tularensis* subsp. *tularensis* FSC033	F. *tularensis* subsp. *novicida* GA99-3548	*F. tularensis* subsp. *novicida* GA99-3549
**Length (Mb)**	1.89	1.87	1.85	1.86	1.90
**GC Content (%)**	32.10	32.07	32.17	32.34	32.23
**Total ORFs**	1,764	1,745	1,715	1,705	1,720
**Average ORF Length (nt)**	916	953	964	986	1000
**Protein Coding ORFs**	1487	1510	1514	1649	1661
**Disrupted ORFs**	277	235	201	56	59
**Percent intact ORFs (%)**	84	87	88	97	97
**Proteins of Unknown Function**	427	604	598	521	537
**Pathogenicity Islands**	2	2	2	1	1
**IS elements**	113	110	74	9	24
**tRNA**	27	32	27	36	30
**rRNA**	5	5	4	7	6
**ncRNA**	4	4	4	4	4
**Coverage**	10.01×	10.31×	10.48×	9.88×	9.88×
**Assembly Size (Mb)**	1.89	1.87	1.85	1.86	1.90
**Total Contig Length (Mb)**	1.89	1.86	1.84	1.85	1.90
**Scaffolds**	21	9	8	5	9
**Scaffold N50 (Kb)**	245.19	488.1	387.07	554.42	298.79
**Contigs**	31	19	15	18	15
**Contig N50 (Kb)**	116.80	293.90	295.52	238.33	209.54
**%Q40**	97.53	98.76	98.76	98.78	98.93
**EndSequenced Fosmids**	27,832	24,312	24,672	26,882	23,927

### IS Element-Based Genome Rearrangement Events in Type B Strains

**Figure 2:**
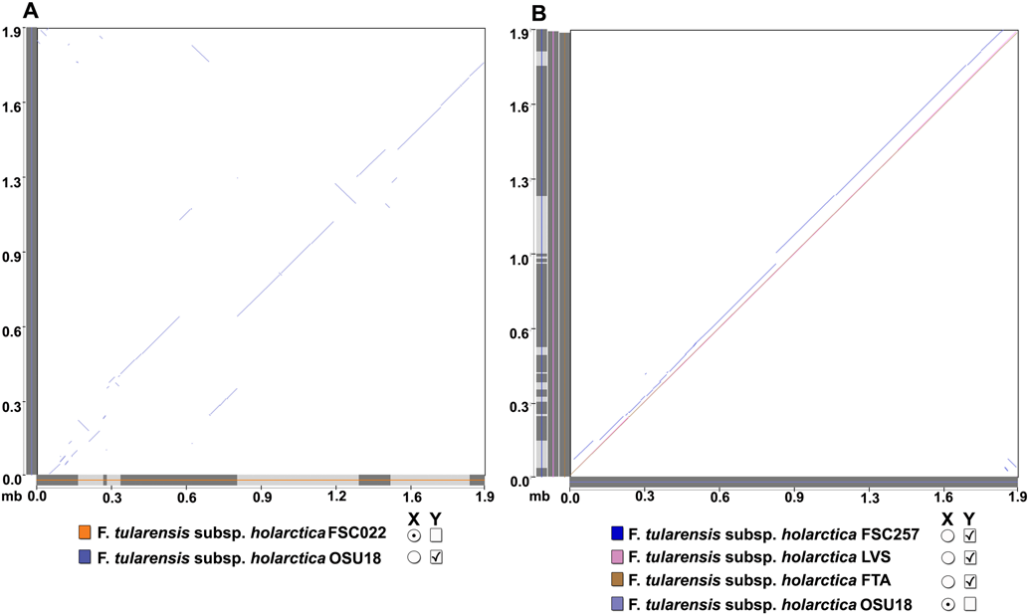
Dotplot comparison between the *Francisella tularensis* subsp. *holarctica* strains showing the occurrence of significant rearrangement events. Whole genome alignments and dotplot comparisons between Type B strains of *Francisella*: (A) *F. tularensis* subsp. *holarctica* OSU18 and *F. tularensis* subsp. *holarctica* FSC022 (reference genome) and (B) *F. tularensis* subsp. *holarctica* LVS, *F. tularensis* subsp. *holarctica* OSU18 (reference genome), *F. tularensis* subsp. *holarctica* FSC257, *F. tularensis* subsp. *holarctica* FTA. Alignments were filtered for overlap percentages greater than or equal to 90%. Dotplot (B) shows a nearly linear, overlapping alignment between all of the Type B strains of the main *holarctica* lineage (not all of the strains of the Type B radiation lineage are shown for clarity), with the notable exception of comparisons between the *F. tularensis* subsp. *holarctica* OSU18 and *F. tularensis* subsp. *holarctica* FSC257 strains. In contrast, numerous rearrangements are evident from comparisons between the *F. tularensis* subsp. *holarctica* FSC022 *japonica* strain and all of the other Type B strains of the main *holarctica* lineage (A) (only comparison to the *F. tularensis* subsp. *holarctica* OSU18 strain is shown here).

Extensive rearrangements have been characterized in *Francisella* and are known to have occurred from comparing different subspecies (e.g., OSU18 vs SCHU S4) [26], as well as from comparing different clades of the *tularensis* subspecies Type A (WY96-3418 vs SCHU S4) [10]; (reviewed in [25]). Here, we provide evidence for the occurrence of large-scale genome rearrangements from whole-genome comparisons between the more ancestral FSC022 *japonica* strain and other Type B strains (Figure 2A) as well as from comparisons within the Type B-radiation lineage (Figure 2B).

In order to gain further insight into the possible mechanisms underlying rearrangements in the Type B strains, we have analyzed IS element content. In general, our analysis of the different classes of IS elements in the five new *Francisella* sequences agrees with what is known of the different *Francisella* subspecies (Table 2) and the conservation profile and genomic context of the IS elements in *Francisella* subspecies are also consistent with present models of phylogenetic relationships of the *Francisella* genus [25] (Table 2) . In agreement with earlier reports, we also find noticeable copy number differences between ISFtu elements when comparing the *F. tularensis* subsp. *novicida* and *F. philomiragia* subsp. *philomiragia* species with the *F. tularensis* subsp. *tularensis* (Type A) or *F. tularensis* subsp. *holarctica* (Type B) subspecies. Specifically, the *F. tularensis* subsp. *novicida* and *F. philomiragia* subsp. *philomiragia* genomes contain predominantly ISFtu2 and ISFtu3 elements, respectively. However, the genomes of Type A and B strains contain significantly higher numbers of ISFtu1 and ISFtu2 elements in comparison to the other ISFtu types 3–6. The abundance of ISFtu1 and ISFtu2 elements in the *F. tularensis* subsp. *holarctica* genomes suggests that the mechanism mediating genome rearrangements in this subspecies may in part be similar to the rearrangements characterized from comparisons between the Type A and B subspecies [26]. However, ISFtu2 copy number and genetic structural differences between Type A and Type B strains may determine rearrangement potential between different Type B strains (reviewed in [25]).

**Table 2:** IS Element Summary Table.

*Francisella* Subspecies Type	Subtype_Strain	ISFtu1 (IS630 family)	ISFtu2 (IS5 family)	ISFtu3 (ISNCY family)	ISFtu4 (IS982 family)	ISFtu5 (IS4 family)	ISFtu6 (IS1595 family)	Total
*tularensis* (Type A)(highly virulent)								
	A.I_FSC033	55	17	3	1	1	4	81
	A.I_SchuS4	53	16	3	1	1	4	78
	A.I_FSC198	53	16	3	1	1	4	78
	A.II_WY96	56	18	3	1	1	4	83
*holarctica* (Type B)(less virulent)								
	FSC257	64	44	2	2	1	3	116
	FSC022	58	57	2	2	1	3	123
	OSU18	66	42	2	2	1	3	116
	LVS	59	46	2	2	1	3	113
	FTA	59	42	2	2	1	3	109
	FSC200	59	41	2	2	1	3	108
*novicida* (rarely human pathogenic)								
	GA99-3548	1	7	0	2	0	1	11
	GA99-3549	0	21	3	2	0	3	29
	U112	2	20	4	1	0	2	29
*philomiragia* (human nonpathogenic)								
	ATCC25017	0	1	4	1	0	2	8

Previous studies have characterized distinct biochemical characteristics, as well as differences in erythromycin sensitivity, among strains of the Type B clade (reviewed in [12]). The *F. tularensis* subsp. *holarctica* is comprised of strains isolated from different geographical regions in the Northern Hemisphere. *F. tularensis* subsp. *holarctica* is, therefore, endemic to different continents with diverse ecologies. As a result, many variations occur in local transmission cycles, environments, and hosts; And give rise to distinctive biochemical and epidemiological traits between certain isolates of the subspecies. For example, the FSC022 *japonica* isolate is recognized as a biovar variant [27] separable from the other Type B strains. In addition, Lagomorphs (rabbits, hares) have been reported to be the predominant natural reservoir for *F. tularensis* in North America, Europe and Japan. Whereas isolates from the former Soviet Union, Sweden, and Norway also inhabit lemmings in addition to other small rodents as a natural reservoir [28]. Furthermore, the finding that Type B strains are more resistant to ingestion by water borne ciliates is consistent with the suggestion that Type B strains are able to survive in aquatic environments [29],[30]. Although further subdivisions of the subspecies *holarctica* have been proposed, very few phenotypic attributes have been demonstrated to formally support this and in the absence of comparative genomic evidence, phylogenetic subdivisions of the *F. tularensis* subsp. *holarctica* has not been formally established or recognized (reviewed in [12]).

**Figure 3:**
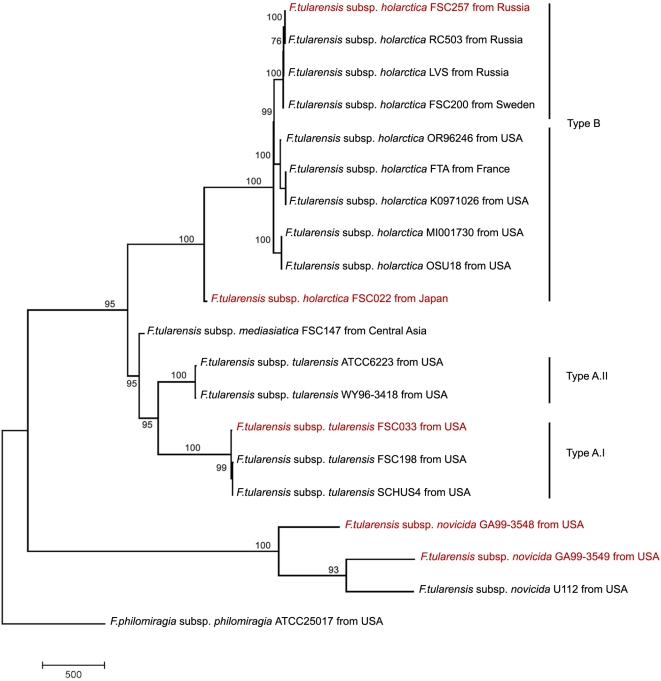
Phylogeny of 20 *Francisella* strains by analysis of Genome-wide SNP sequences and inferred using Maximum Parsimony method. The evolutionary history of 20 Francisella strains was inferred from analysis of genome-wide SNP sequences. Genome-wide SNPs at least 20 bp apart were selected for further analysis using MEGA4 software. The tree is drawn to scale, with branch lengths calculated using the average pathway method and are in the units of the number of changes over the whole sequence. *Francisella* strains sequenced at the Broad Institute are in red. The differentiation of *F. tularensis* subsp. *novicida* predated differentiation of the more pathogenic *F. tularensis* subsp. *tularensis* and *F. tularensis* subsp. *holarctica* subspecies from a common ancestor. Our data indicates differential rates of evolution along the Type A.II and Type A.I lineages, as evident from the branch lengths. The Type B FSC022 strain diverged prior to the radiation of the main *holarctica* group and also has a greatly reduced branch length, consistent with a much slower rate of evolution. The *F. tularensis* subsp. *mediasiatica* FSC147 strain is phylogenetically more closely related to the Type A.II lineage even though its pathogenicity is characteristic of Type B strains.

The occurrence of rearrangement events in the Type B lineage emphasizes the phylogenetically distinct nature of the FSC022 strain and an appreciation of the impact that genetic decay likely has on the differentiation of *F. tularensis* subsp. *holarctica*. In this regard, it is important to emphasize the more recent emergence of the *F. tularensis* subsp. *holarctica* subspecies in comparison to other *Francisella* clades. FSC022 represents a more distinct lineage that predated strains presently experiencing the highest levels of genome decay in comparison to other subspecies, as evident by the abundance of pseudogenes (Table 1, and Tables 3–6). Although we identify genetic differences specific to the Type B strain isolates from Sweden and Russia (FTT0023c in Table 4 and FTT0159c in Table S1d), significant changes over the whole sequence are not evident (Figure 3).

**Table 3:** Phylogenetic Differentiation Associated with Gene Gain and Loss.

Presence of gene (Intact, disrupted, or absent) and predicted protein family name subcategory	Subspecies strains	LocusID (SchuS4 Reference)	Description of predicted protein product (gene name)
**Intact**	**Type A strains only**	FTT0496	hypothetical protein
		FTT0677c	hypothetical protein
		FTT0939c	adenosine deaminase (*add*)
		FTT1068c	hypothetical protein (A.I subspecies specific)
		FTT1080c	hypothetical membrane protein
		FTT1122c	hypothetical lipoprotein
		FTT1766	O-methyltransferase
		FTT1791	hypothetical protein
**Intact**	**Type A and B strains only**	FTT0524	hypothetical protein
		FTT1172c	cold shock protein (*csp*)
**Intact**	**Type A and B strains and disrupted or absent in at least one non- or rarely-human pathogenic strain**	FTT0755	hypothetical membrane protein
		FTT1011	hypothetical protein
		FTT1580c	hypothetical protein
**Intact**	**Type B strains only**	FTT1175c	hypothetical membrane protein
**Disrupted or absent**	**Type A strains only**	FTT0214	pseudogene, transporter protein
		FTT0514	L-lactate dehydrogenase (*lldD1*)
		FTT0529c	DNA polymerase IV, devoid of proofreading, damage inducible protein P (*dinP*)
		FTT0652c	ferritin-like protein (*ftnA*)
		FTT1378	pseudogene, hypothetical protein
		FTT1429c	pseudogene, hypothetical protein
		FTT1516c	mercuric reductase (*merA*)
		FTT1619	pseudogene, acetyltransferase
		FTT1661	thiopurine S-methyltransferase (*tmpT*)
		FTT1768c	chitinase
		FTT1786	pseudogene, hypothetical protein
		FTT1793c	aminopeptidase N (*pepN*)
		FTT1799c	pseudogene, hypothetical protein
Transporters: The ATP binding Cassette (ABC) Superfamily		FTT0276c	cyclohexadienyl dehydratase precursor
		FTT0445	ABC transporter, ATP-binding component
Transporters: The Major Facilitator Superfamily (MFS)		FTT0657	major facilitator superfamily (MFS) transporter
		FTT0775c	major facilitator superfamily (MFS) transporter (*bcr2*)
		FTT1380	major facilitator superfamily (MFS) transporter
		FTT1618	major facilitator superfamily (MFS) transporter
**Disrupted**	**Type B strains only**	FTT0178c	30S ribosomal protein S6 modification protein-related protein (*rimK*)
		FTT0221	acid phosphatase precursor (*acpA*)
		FTT0544	phosphonoacetate hydrolase (*phnA*)
		FTT0553	hypothetical protein
		FTT0568	hypothetical protein
		FTT0747c	hypothetical protein
		FTT0783	Arylsulfatase (*ars*)
		FTT0786	hypothetical protein
		FTT0846	deoxyribodipyrimidine photolyase
		FTT0898c	hypothetical protein
		FTT0902	hypothetical protein
		FTT0949c	hypothetical membrane protein
		FTT1007c	hypothetical protein
		FTT1109	choloylglycine hydrolase family protein
		FTT1171c	DNA-methyltransferase, Type I restriction-modification Enzyme subunit M (*hsdM*)
		FTT1202	transcriptional regulator lysR family
		FTT1267	transcriptional regulator lysR family
		FTT1293c	hypothetical protein , sua5_yciO_yrdC family protein
		FTT1383	Sun protein
		FTT1413	aminotransferase
		FTT1428c	acetyltransferase
		FTT1591	lipoprotein
		FTT1623c	hypothetical protein
		FTT1625c	hypothetical protein
		FTT1796c	hypothetical protein
Transporters: The ATP binding Cassette (ABC) Superfamily		FTT0017	ABC transporter ATP-binding protein for toxin secretion
		FTT0125	oppD, oligopeptide transporter, subunit D
		FTT0475	the small conductance mechanosensitive ion channel (MscS) family transporter
		FTT1775c	the chloride channel family transporter
Transporters: The Major Facilitator Superfamily (MFS)		FTT0129	major facilitator superfamily (MSF) transporter
		FTT0487	major facilitator superfamily (MSF) transporter
		FTT0488c	major facilitator superfamily (MSF) transporter
		FTT0671	major facilitator superfamily (MSF) transporter
Transporters: Proton-dependent oligopeptide transport (POT) family		FTT0651	proton-dependent oligopeptide transport (POT) family protein
		FTT1005c	proton-dependent oligopeptide transport (POT) family protein (*yhiP*)
**Disrupted or absent**	**Type A and Type B strains only**	FTT0262	hypothetical lipoprotein
		FTT0495	hypothetical protein
		FTT0706	(*glk1*), glucose kinase
		FTT0865	pseudogene, hypothetical protein
		FTT0883	pseudogene, alcohol dehydrogenase
		FTT1577	hypothetical protein
**Disrupted or absent**	**Type B strains and closely related rarely-human pathogenic ** ***F. tularensis*** ** subsp. ** ***mediasiatica*** ** FSC147**	FTT0095	hypothetical protein
		FTT0122	(*oppA*), oligopeptide transporter, subunit A * intact in strain FSC022
		FTT0177c	acetyltransferase
		FTT0223c	hypothetical protein (*ybgL*)
		FTT0464	(*ansB*), periplasmic L-asparaginase II precursor
		FTT0673c	hypothetical protein
		FTT0829c	aspartate∶alanine antiporter
		FTT0850	hypothetical protein
		FTT0864c	transcriptional regulator lysR family
		FTT0911	hypothetical protein
		FTT0961	(*mdaB*), modulator of drug activity B
		FTT0995	major facilitator superfamily (MSF) transporter
		FTT1119	transcriptional regulator lysR family
		FTT1266c	(*yhhW)*
		FTT1285c	transcriptional regulator lysR family
		FTT1592c	pseudogene, hypothetical protein
		FTT1645	major facilitator superfamily (MSF) transporter
		FTT1703	hypothetical protein
		FTT1781c	hypothetical protein
Membrane proteins		FTT1426c	hypothetical membrane protein
		FTT1626c	hypothetical membrane protein

**Table 4 ppat-1000459-t004:** Phylogenetic Differentiation Associated with Gain and Loss of Genes with Sequence Similarity to T3SS Effectors.

**Disrupted or absent**	**Type A and/or B strains only**	FTT0023c	lipase/acyltransferase
		FTT1524c	ATP-dependent helicase (*hrpA*)
**Disrupted or absent**	***F. tularensis*** ** subsp. ** ***mediasiatica*** **, Type A and B strains only**	FTT0612	hypothetical protein (present in three copies in novicida strains)
**Present**	**All subspecies strains**	FTT0211c	outer membrane lipoprotein
		FTT0393	methionine aminopeptidase (*map*)
		FTT0541c	haloacid dehalogenase (*yqaB*)
		FTT0659	DNA recombination protein (*rmuC*)
		FTT0910	hypothetical protein
		FTT1132c	glycerophosphoryl diester phosphodiesterase (*glpQ*)
		FTT1156c	Type IV pilin multimeric outer membrane protein (*pilQ*)
		FTT1268c	chaperone protein (*dnaJ*)
		FTT1376	acyl carrier protein (*acpP*)
		FTT1512c	chaperone protein (*dnaJ1*)
		FTT1671	riboflavin biosynthesis protein (*ribD*)

**Table 5 ppat-1000459-t005:** Phylogenetic Differentiation Associated with Gain and Loss of Genes with Sequence Similarity to T4SS Components.

**Disrupted or absent**	***F. tularensis*** ** subsp. ** ***mediasiatica*** **, Type A, and B strains only**	FTT0046	magnesium chelatase family protein (*comM*)
		FTN_1756	bacterioferritin comigratory protein (*bcp*)
**Disrupted or absent**	***F. tularensis*** ** subsp. ** ***mediasiatica*** ** only**	FTT1797c	peptide methionine sulfoxide reductase (*msrA2*)
**Disrupted or absent**	**Type A strains only**	FTT0542	peroxiredoxin (alkyl hydroperoxide reductase subunit C) (*prdX/ahpC*)
**Present**	**All subspecies strains**	FTT0458	stringent starvation protein A regulator of transcription (*sspA*)
		FTT0557	hypothetical protein ahpC/TSA family
		FTT0623	trigger factor (TF) protein (peptidyl-prolyl cis/trans isomerase)
		FTT0628	peptidyl-prolyl cis-trans isomerase D
		FTT0633	membrane protease subunit (*hflK*)
		FTT0634	membrane protease subunit (*hflC*)
		FTT0832	FKBP-type 16 kDa peptidyl-prolyl cis-transisomerase (*fkpB*)
		FTT0878c	peptide methionine sulfoxide reductase (*msrB*)
		FTT1186	SsrA (tmRNA)-binding protein (*smpB*)
		FTT1422	SM-20-related protein
		FTT1725c	protein-L-isoaspartate O-methyltransferase (*pcm*)

**Table 6:** Phylogenetic Differentiation Associated with Gain and Loss of Genes Regulating Competence (E values>1e-10).

**Disrupted or absent**	**At least one strain of a human pathogenic subspecies**	FTT0046	magnesium chelatase family protein (*comM*)
		FTT0179	DNA internalization-related competence protein (*rec2*), *Not present in Type B, OSU18 strain
		FTT0830c	DNA uptake protein (*dprA*)
		FTT1301c	amidophosphoribosyl-transferase (similar to *comF*)
**Present**	**All subspecies strains**	FTT1057c	fimbrial biogenesis and twitching motility protein (*fimB*) (*In novicida subspecies, present only in U112)
		FTT1156c	Type IV pilin multimeric outer membrane protein

### Comparative Analysis of Human Pathogenic and Non-Pathogenic Strains of *Francisella tularensis* Subspecies

**Phylogeny of 20 *Francisella* strains by analysis of genome-wide SNP sequences.** Phylogenetic studies of genomewide SNP sequences from 20 *Francisella* strains show the population structure and subspecies divergence of *Francisella* and overall, the relationships are consistent with what has been previously reported (Figure 3) [4],[11],[31]. In agreement with these studies, it is evident from our phylogenetic analysis that the differentiation of *F. tularensis* subsp. *novicida* predated differentiation of the more pathogenic *F. tularensis* subsp. *tularensis* and *F. tularensis* subsp. *holarctica* subspecies from a common ancestor (Figure 3). We also find that the branch length leading to *F. tularensis* subsp. *tularensis* Type A.II strains is shorter than to the *F. tularensis* subsp. *tularensis* Type A.I strains, suggesting differential rates of evolution along these two lineages. The *F. tularensis* subsp. *holarctica* FSC022 strain diverged basally from the main *holarctica* lineage prior to the radiation of the main *holarctica* group and also has a greatly reduced branch length consistent with a much slower rate of evolution. The *F. tularensis* subsp. *mediasiatica* FSC147 strain is phylogenetically more closely related to the Type A.II subspecies (Figure 3, and Larsson *et al.*, submitted), however, cases of human infection are very rare. Interestingly, findings that human mortality results from infection with strains of the Type A subtype is indicative of host adaptation mechanisms possibly leading to an attenuation of virulence in both the *F. tularensis* subsp. *mediasiatica* and the *F. tularensis* subsp. *holarctica* (Type B) subspecies (reviewed in [12]).

#### Phylogenetic differentiation associated with genetic acquisition and decay

Due to the similarity of their overall genomic sequences (Figure 1), it has been proposed that subspecies and strain-to-strain pathogenicity differences in *Francisella* are most likely the result of smaller-scale polymorphisms found proximal to predicted breakpoints of genomic rearrangement events [17]. Previous analysis of a transposon generated *F. tularensis* subsp. *novicida* mutant library identified 396 candidate essential genes required for growth *in vitro* [32]. A recent study using this same transposon library to perform a negative-selection screen in a mouse model identified 125 candidate virulence genes required for infection of the lung, liver and spleen [33]. A different *in vivo* screen, identified 164 genes important for *F. tularensis* subsp. *novicida* virulence in mice [34]. The *F. tularensis* subsp. *novicida* strain is commonly used as a model organism to assay general *Francisella* subspecies virulence due to its pathogenicity *in vitro* and in small animal models. However, the very rare occurrence of human infection from *F. tularensis* subsp. *novicida* provides a need for studies that identify genes involved in regulating growth and virulence by comparative approaches across subspecies [35]. Other, more comparative, studies have identified genes that are either absent or disrupted (pseudogenes) in certain *Francisella* subspecies [9],[17],[36]. A recent comparative study of the Type A, Schu S4, Type B LVS, and *F. tularensis* subsp. *novicida* U112 strains reported 41 genes unique to the Type A and B strains, with most of these encoding proteins of unknown functions [17].

Our analysis of 20 *Francisella* genomes has identified a subset of ∼500 coding sequences that are disrupted in different subspecies and included are genes known to encode protein products involved in metabolic pathways, intercellular transport, secretion, the Type I restriction-modification defense system, transcription, signalling (ie. two component systems), and many hypothetical proteins with unknown functions (listed in Tables 3–6 and Table S1a–h). Only 112 genes identified in our comparative analysis have also been previously characterized by other studies using an individual strain, or a small subset of strains, as potential candidates important for *Francisella* virulence (Table S2) [17], [32]–[37]. Our analysis of many additional strains of each subspecies has produced a more comprehensive list of gene functions unique to the human pathogenic Type A and B strains in comparison to previous studies [9],[17],[36]. Our studies also provide novel characterization of candidate genes regulating pathogenesis in the *F. tularensis* subsp. *mediasiatica* subspecies. Some of which exhibit sequence similarity to members of the Type III and IV secretion systems that although known to play a role in mediating bacterial pathogenicity, are not intact or known to be functional in *Francisella* subspecies (Tables 4 and 5). As a result, comparison of highly, moderately, and rarely/non- virulent strains has provided a more comprehensive list of potential virulence factors mediating human infection by *Francisella* (Tables 3–6 and Table S1a–h).

#### Gene functions specific to human-pathogenic *Francisella* strains

In total, we have identified fourteen genes that are intact only in the *Francisella* human pathogenic strains (Type A and Type B strains), eight of these are specific to the more virulent Type A strains and are either absent or disrupted in other subspecies (Table 3). We have found that previously identified genes encoding hypothetical proteins and the O-antigen cluster in Type A and B strains are also intact in the *F. tularensis* subsp. *mediasiatica* FSC147 strain, and this O-antigen cluster is distinct from the cluster type characterized in isolates of *F. tularensis* subsp. *novicida* and *F. tularensis* subsp. *philomiragia* [17].

With the exception of *add* (FTT0939), an adenosine deaminase and a gene (FTT1766) encoding O-methyltransferase, all of the eight genes specific to Type A strains are predicted to encode either proteins of unknown function or potential membrane proteins, (Table 3). These ORFs are intact in all of the Type A strains, but disrupted in strains of the Type B subtype as well as in strains of the non-human pathogenic subspecies. In addition, we note the presence of a gene (FTT1068c) encoding a hypothetical protein that is intact specifically in the Type A.I clade and was previously identified by Beckstrom-Sternberg SM *et al.* [10]. All of the Type A specific loci have been previously reported [17], but not previously identified to be disrupted in *F.tularensis* subsp. *mediasiatica*. There is a collection of genes that are intact only in Type A and/or B strains and although their functions are not known, a few are predicted to encode membrane proteins (Table 3). In addition, we find that a gene (FTT0604) encoding a CPA1 family antiporter has an in-frame stop upstream of a transmembrane domain in Type A strains that is not present in Type B strains (data not shown) [17]. This large protein family is highly conserved, and functionally characterized to catalyze Na+: H+ exchange. Although much less is known regarding their primary physiological function in bacteria, there is evidence that CPA transport systems act to regulate cytoplasmic pH and mediate electrophile resistance [38].

The metabolic pathways and growth requirements for the Type A Schu S4 strain have been characterized previously and include 350 enzymes involved in 137 predicted pathways of small molecule metabolism [9]. These studies also established that the growth requirements for this strain include a supplemental supply of 14 essential amino acids as well as cysteine, for sulfate assimilation. Our comparative analysis confirms that most, if not all, pathways for amino acid synthesis seem to be inactivated in Type A and B strains, as well as in the *F. tularensis* subsp. *mediasiatica* FSC147 strain (Table S1a; [9],[17]. Consistent with previous studies, we find that most of the purine metabolism pathway is interrupted, however, no subspecies-specific gene inactivation was found in pyrimidine metabolism pathway. The significance of a Type A specific intact adenosine deaminase ORF is unknown [17], however, studies have characterized the important role of adenosine editing in regulating survival of parasites, which like *tularensis*, are also spread through insect vectors (ie. Malaria, Leishmania, T. brucei) and speculate a role for adenosine deaminases in regulating T. brucei variant surface glycoproteins ([39]). Of particular interest is its role as a metabolic trigger response, converting adenosine to inosine, which promotes the opportunistic pathogenicity of *Pseudomonas aeruginosa* [40]. Genes encoding components of the pentose phosphate pathway and involved in glycolysis were also found to be disrupted in strains of the Type A and B subtypes, as well as in the *F. tularensis* subsp. *mediasiatica* FSC147 strain (Table S1a).

#### Identification of genes in decay and potential regulators of attenuation in the *Francisella* type B strains and closely related human non-pathogenic *F. tularensis* subsp. *mediasiatica* genome

**Figure 4:**
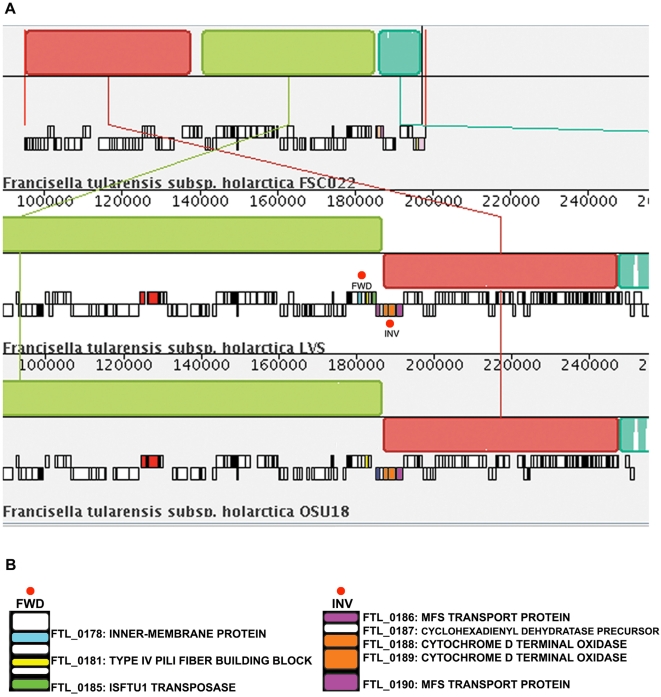
Multiple alignment of *Francisella* genomes of the Type B lineage identifies conserved sequence regions with rearrangements; pseudogenes are found proximal to predicted breakpoints. A comparison of genome rearrangement patterns between the more ancestral *F. tularensis* subsp. *holarctica* FSC022 japonica strain and representative strains of the main *holarctica* group (*F. tularensis* subsp. *holarctica* LVS and *F. tularensis* subsp. *holarctica* OSU18) was done using MAUVE (A). MAUVE uses an anchored alignment algorithm that permits reordering of the alignment anchors for identification of rearrangements. Colored Local Collinear Blocks (LCBs) are regions of sequence alignment that are free of rearrangements. Each LCB is defined by the anchor regions or predicted sites of rearrangement. A default LCB cutoff of 175, and a filtering for larger blocks containing 10 Kb or larger was done in Mauve. Sequence inversions are denoted by differential positioning of the LCBs relative to a reference axis. A zoomed-in section of the whole-genome alignment is shown so that the annotated ORFs are visible (black outlined boxes). The small red ORFs are rRNA genes. ORFs proximal to predicted rearrangement breakpoints (red circles) have been color coded and labeled (FWD, INV). A summary of the predicted protein products for these genes is provided in (B). These include genes that have been identified as being either disrupted or absent in the *F. tularensis* subsp. *holarctica* subspecies.

**Figure 5:**
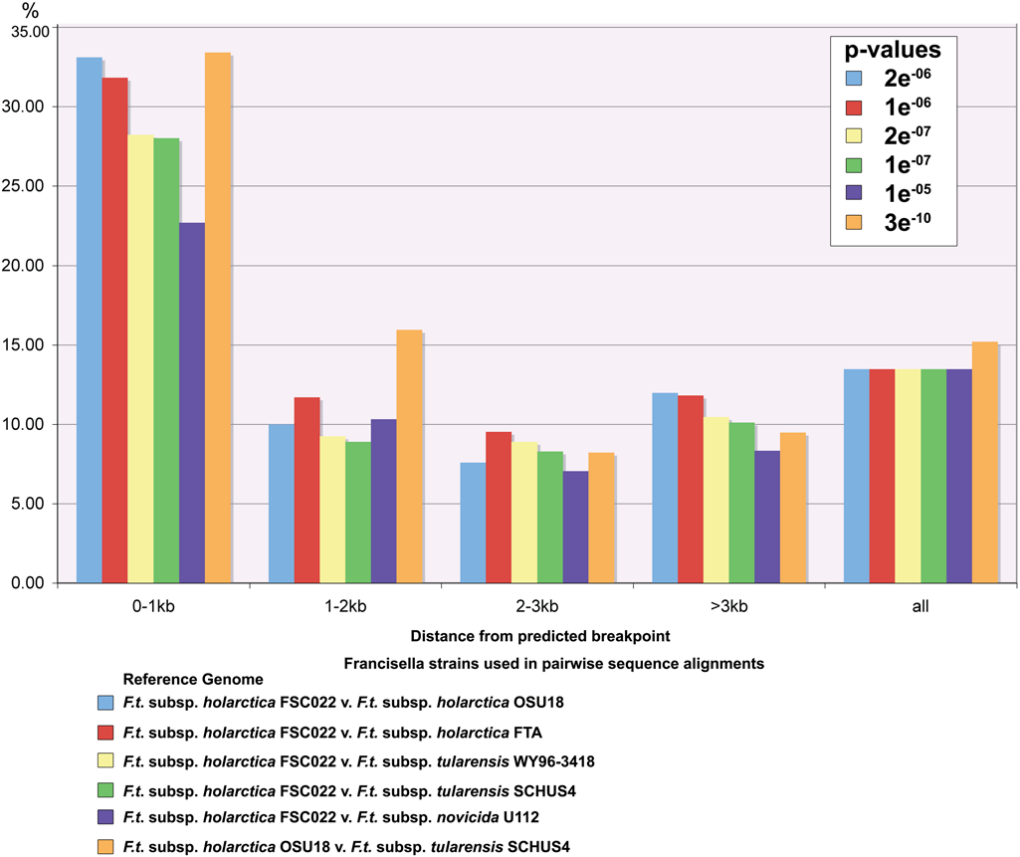
Comparisons of pairwise sequence alignments provides evidence of pseudogene enrichment within 1 kb of predicted breakpoints in Type B strains. Pairwise sequence alignments using *F. tularensis* subsp. *holarctica* (Type B) strains as a reference were further analyzed for evidence of gene decay proximal to predicted breakpoint sites. Total ORF counts were used to determine the percentage of pseudogenes (y-axis) relative to the distance from the predicted breakpoint (x-axis). Pseudogene enrichment was determined using Fisher's Exact Test to derive p-values. Our findings show the highest percentages of pseudogenes are found within 1 kb of the predicted breakpoints indicating that genome rearrangement events have likely promoted gene decay in strains of the Type B lineage.

Our finding that there are a significant enrichment of pseudogenes within 1 kb of the FSC022 predicted breakpoints indicate that genome rearrangement events have likely promoted genetic decay in the Type B lineage (Figures 4 and 5). Similarly, we also report the occurrence of rearrangement events when comparing the *F. tularensis* subsp. *mediasiatica* FSC147 strain with the other *Francisella* subspecies strains (Figure S1). Comparative analysis of these newly available strains with highly pathogenic Type A subspecies strains and more distant rare/non-pathogenic *novicida* and *philomiragia* strains has provided new insight into how gene decay in different gene functional classes may have contributed to the attenuation of pathogenicity in Francisella.

*DNA metabolism and oxidative stress*. In agreement with previous studies, we find that genes encoding members of the LysR transcriptional regulator family as well as other proteins involved in transcription, such as acetyltransferases, are specifically disrupted or absent in Type B strains [17]. Not previously known, was the inactivation of these genes in the closely related non-human pathogenic *F. tularensis* subsp. *mediasiatica* FSC147 strain (Table 3). The range of known LysR transcriptional regulator targets includes genes whose roles include niche adaptation and virulent responses [17],[41]. The functional importance of the LysR transcriptional family in mediating pathogenicity, and their notable decay in the *F.t. holarctica* and *F.t. mediasiatica* subspecies lends support to the notion that they may be responsible for attenuation of virulence.

Also of interest is the subspecies specific loss of the *hsdM* gene (FTT1171c) in the more moderately human pathogenic Type B strains, which encodes a DNA methyltransferase component of the Type I restriction-modification defense system (Table 3). Previous studies have established that the Type I restriction-modification defense system regulates resistance of horizontal gene transfer in *S. aureus* [42]. And a recent study reports the presence of a strong restriction barrier in the *F. tularensis* subsp. *novicida* subspecies that is mediated by numerous restriction/modification systems, which have been lost during the evolution of the human pathogenic subspecies [35].

*Secretion systems*. Generally, the cell envelope of gram-negative bacteria is composed of an outer membrane, peptidoglycan cell wall, periplasm and an inner membrane. Secretion systems acting across the cell envelope mediate transfer of virulence factors, and are therefore fundamental regulators of bacterial pathogenesis [43]. Interestingly, early studies that identified virulence factors characterized a particular set of genes homologous to components of the Type III secretion system in *Yersinia* . These genes are frequently associated with pathogenicity islands present in animal pathogens as well as in plants [44],[45]. In addition, two Type III effector genes of the gram-negative pathogenic bacteria that causes blight in rice have been shown to mediate host transcription factor induction [46].

Previous studies have reported the presence of many components of different secretion systems in *F. tularensis* [25],[47]. Overall, our analysis of the recently sequenced *F. tularensis* genomes is consistent with previous characterization of the genes (and psuedogenes) that encode components of the Twin Arginine Translocation (TAT), and secretion systems of Type I (T1SS), Type II (T2SS), Type V (T5SS), and VI (T6SS) (Tables 4–6 and Table S1c,d,f) [44],[48],[49] in the *F. tularensis* subsp. *tularensis* and *F. tularensis* subsp. *holarctica* strains. There is presently no existing evidence to support the presence of intact Type III (T3SS) or Type IV (T4SS) secretion systems, or their functional role in *Francisella*, with the exception of the Type IV pilus biogenesis system. Although this system shares components of the T3SS, it is primarily associated with the T2SS [9],[25],[47],[49] (Table 4).

The Type III secretion system (T3SS) mediates transfer of virulence factors into host cells, and the T4SS may mediate transfer of DNA or virulence factors into eukaryotic target cells. Both systems are major pathogenicity factors in other bacteria [48],[50],[51]. And the secreted T3SS effectors have been shown to be important contributors to virulence in bacterial pathogenesis [48]. Using *P. aeruginosa* T3SS component proteins and a repertoire of effector proteins to query *Francisella*, similar to the approach of Tobe, *et. al.* [48], we identified several homologs of known T3SS effectors (Table 4). This same approach did not identify any significant hits (e<1^e-5^) to *Agrobacterium tumefaciens* C58 T4SS proteins that were queried against the *Francisella* genomes [52],[53]. However, we did identify a small number of *Francisella* genes, using the KEGG system, which were assigned to T4SS pathways (Table 5).

Interestingly, the genes with similarity to effectors of the T3SS and components of the T4SS systems are present in *F. tularensis* subsp. *novicida* and *F. philomiragia*. subsp. *philomiragia*, however, there are four that are either present as pseudogenes or absent in Type A and/or Type B strains and one of these is also disrupted in *F. tularensis* subsp. *mediasiatica*. Two genes similar to T3SS effectors are present as pseudogenes specifically in Type A.II and Type B strains isolated from the Russian and Sweden regions (Table 4). One of these genes, *hrpA* (FTT1524c), encodes for an ATP-dependent helicase homologous to the *E. coli* HrpA DEAH-box RNA helicase previously shown to be involved in mRNA processing of an operon involved in fimbrial biogenesis [54], and the other gene, (FTT0023), encodes for a lipase/acyltransferase. In *novicida* strains, three genes (FTN_1069, FTN_1070, FTN_1071) are similar to the T3SS effector, OspD3_Sflx. These genes are truncated in Type A (FTT0612), Type B, and *mediasiatica* subspecies strains.

Type IV pilus secretion systems form a trans-envelope channel and an extracellular pilus structure. Type IV pili are known to mediate bacterial adhesion and twitching motility, and possibly other cellular functions necessary for bacterial growth and pathogenesis [55]. Genes required for Type IV pili biogenesis have been previously identified in *Francisella tularensis* genomes and several of these encode components shared by the T3SS (ie. flagella), or are homologous to genes also required for T2SS [9],[25]. Consistent with previously characterized Type B strains, we identified an early in-frame stop in the *pilT* gene (FTT0088, T2SS) present in FSC022 and FSC257. This gene is also disrupted in the *F. tularensis* subsp. *mediasiatica* FSC147 strain, indicating that this genetic disruption occurred after the phylogenetic split between the Type A and *F. tularensis* subsp. *mediasiatica*/Type B *Francisella* strains ([56], Table S1f). FTT0861c, encoding a Type IV pili fiber block protein, has a large internal deletion and frame-shift in all of the Type A strains and in the FSC257 and FSC200 Type B strains. All of the Type B strains isolated from Russia and Sweden have a 5-bp deletion, which restores the reading frame without introducing an internal stop codon (data not shown). The prepilin peptidase encoded by the *pilD* (FTT0683c) gene has an in-frame stop near the C-terminus in all Type A and B strains, making them 9aa shorter. The impact of this change with regards to pathogenetic capacity of different *Francisella* strains is not known, however, *Legionella pneumophila pilD* mutants are known to be greatly reduced in virulence [57]. In addition, we report that the *pilA* gene in all of the *novicida* strains is a single gene corresponding to a merge of FTT0888, FTT0889 and FTT0890, each about the size of *pilA* genes in other bacterial species. Although the biological significance of this merging of *pilA* genes is unknown, previous studies have established a functional importance of gene fusions in mediating virulence. For example, the fusion of the partial ORFs, FTT0918 and FTT0919, has been shown to be a significant contributing factor to virulence attenuation in the FSC043 strain [58]. Recent studies have also characterized the presence of fibres resembling type IV pili on the surface of the LVS and demonstrated the importance of *pilA* and the other genes of the type IV pilus biogenesis system in mediating *F. tularensis* virulence in mice [59],[60]

Most of the genes identified by their similarity to known T4SS genes are present in all of the *Francisella* subspecies. Of interest are two genes, FTT1797c and FTT0542; the former is disrupted only in the *F. tularensis* subsp. *mediasiatica* FSC147 strain and the latter is specifically disrupted in the *F. tularensis* subsp. *tularensis* subspecies. The disruption of a gene (FTT1797c), encoding a peptide methionine-S-sulfoxide reductase, only in the *F. tularensis* subsp. *mediasiatica* FSC147 strain is notable given emerging evidence that the absence of the MsrA2 enzyme results in decreased bacterial pathogen survival in host organisms [61]. Also of interest is the subspecies specific loss of the gene (FTT0542) encoding the peroxiredoxin oxidative response protein in Type A strains (Table 5).

It is also worth noting that *comM*, a gene disrupted in *F. tularensis* subsp. *tularensis*, *F. tularensis* subsp. *holarctica*, and *F. tularensis* subsp. *mediasiatica*, has been previously characterized in *Haemophilus influenzae* and is also a highly conserved component of systems regulating competence in gamma-proteobacteria and *E. coli* [62]. Additional loci shown to genetically interact with the *comM* competence pathway were also identified in all of the *Francisella* subspecies, including the *F. tularensis* subsp. *mediasiatica*, *F. tularensis* subsp. *novicida* and *F.philomiragia* subsp. *philomiragia* strains (Table 6).

*The* Francisella *Pathogenicity Island*. The *Francisella* Pathogenicity Island is comprised of a regulated cluster of approximately 19 genes known to regulate intramacrophage growth [18]. Previous studies have classified genes found in the *Francisella* Pathogenicity Island as components of the T6SS [16],[18]. Inactivation of one of these genes, *pdpD*, results in attenuation of *F. tularensis* in mice, and it has also been previously reported that *pdpD* is either absent or present as a strain specific isoform in the Type B subtype [16],[63]. Our computational analysis of ten Type B strains has enabled us to further characterize the *pdpD* disruption. We report that *pdpD* is significantly truncated by an in-frame stop codon in all Type B strains (Table S1f and data not shown). Interestingly, a gene (FTT1348) in the *Francisella* Pathogenicity Island that encodes a hypothetical protein is disrupted in Type B strains as well as in the *F. tularensis* subsp. *mediasiatica* FSC147 strain (Table S1f).

*The Twin Arginine Translocation System*. The Twin Arginine Translocation (TAT) secretion system mediates cytoplasmic membrane transport of folded proteins regulating bacterial pathogenesis. Our analysis using the TatP program identified potential substrates for the (TAT) secretion pathway that were disrupted or absent in the *F. tularensis* subsp. *tularensis* subspecies (Table S1f). The *bcr2* transporter gene (FTT0775c), is specifically disrupted in the Type A.I strains and genes encoding a short-chain dehydrogenase/reductase family protein (FTT0723) and an amino acid transporter (FTT0361) are specifically disrupted in all of the Type B strains. The (FTT0723) gene encoding a short-chain dehydrogenase/reductase family protein and (FTT1510c), which encodes a HAAP family transporter are also disrupted in the *F. tularensis* subsp. *mediasiatica* strain (Table S1f).

*Transporters*. Other major transporter protein families have been identified that regulate growth and virulence in bacteria, and specifically in *Francisella*. For example, the ATP-binding cassette (ABC) as well as the Major Facilitator Superfamily (MFS) transporters have also been shown to be involved in mechanisms of multidrug resistance in both bacteria and fungi [64],[65]. In fact, the largest group of inactivated genes that encode known protein products are classified as members of the MFS transporters (Table 3 and Table S1b,f). We identified a total of 14 MFS and ABC encoding genes that were specifically disrupted or absent in *F. tularensis* subsp. *holarctica* and/or *F. tularensis* subsp. *mediasiatica* (Table 3 and Table S1b). Our comparative analysis also confirmed previous findings regarding specific loss in Type A.II and Type B strains of the FTT0727 and FTT0729 genes that encode components of the DRI/YHIH family ABC exporter, as well as loss of genes encoding members of the OCTN family of ABC transporters, the REG family of ABC transporters and the oppABCDF system [36],[66],[67], Table S1b). The *opp* operon is an important regulator of oligopeptide transport and bacterial growth [68]. We find that two genes, *oppD* (FTT0125) and *oppF* (FTT0126), are separate loci in Type A strains, but a 960 bp internal deletion in Type B strains results in a fused ORF with disrupted *oppD* and *oppF* coding sequences (Table 3 and Table S1g).

*Two Component Systems*. Bacterial Two Component Systems (TCSs) have been found to be important regulators of growth and virulence [69],[70]. We identified three known paired TCSs that exhibit differences between the *Francisella* subspecies: The *ntrXY* regulator (unique to *F. philomiragia*), the *vicRK* system, and the *kdpABCDE* system [9] (Table S1f). Studies in bacterial species other than *Francisella* have provided evidence that the *vicRK* system plays a key role in mediating virulence by actively detecting changes in temperature, oxidative stress and osmotic pressure [71],[72]. We find that two genes of the *vicRK* system are specifically disrupted in strains of the human pathogenic Type A and B lineage (Table S1f) [17],[73].

The *kdp* genes function in a turgor pressure response system that is sensitive to low potassium levels. Interestingly, we find that all of the genes in the *kdp* operon, with the exception of *kdpC* and a small ORF we have identified as *kdpF* (FTT1740c), are differentially inactivated in Type A and B strains, consistent with previous studies [17] as well as in the *F. tularensis* subsp. *mediasiatica* strain, FSC147. Previous work has shown that a transposon mediated disruption of the *kdpD* gene in the *F. tularensis* subsp. *novicida* U112 strain leads to attenuation of virulence in mice [34].

Overall, the biological significance of the subspecies specific genomic differences that we report are largely unknown, except in those cases also identified and verified experimentally by other studies. It is worth mentioning that there are cases, like the *kdpD* gene, where gene loss in other species of bacteria (*M. tuberculosis*) results in increased virulence rather than attenuation [69]. It is known that regulation of highly conserved genetic pathways important in determining virulence and host tropism are dependent upon the overall biology of the organism, and are also influenced by the host environment. In regards to mechanisms of host tropism and *Francisella* infection, much has been gleaned from a recent study suggesting that *F. tularensis* subsp. *holarctica* bacterial dissemination post-infection is accomplished by regulation of dendritic cells migration in mice [74]. Along these lines, it should be mentioned that all *Francisella* subspecies assayed to date are virulent in cultured cells and in animal models, even though the pathogenicity of different subspecies in the human host varies considerably. The *F. tularensis* subsp. *novicida* U112 strain, for example, rarely affects humans even though this strain is highly virulent in animal models. This, together with its genetic tractability, makes *F. tularensis* subsp. *novicida* U112 a commonly used model system in studies of *Francisella* pathogenesis. Although there is much to be learned from these studies, we are still challenged by the inherent limitations that exist in assaying how subspecies genetic differences determine virulence in various host environments, especially in humans.

### Conclusions

We have reported the sequencing of five globally diverse strains of *Francisella* and their comparative analysis with all other publicly available *Francisella* genome sequences, including the geographically restricted and rare *F. tularensis* subsp. *mediasiatica* FSC147 isolate from the Central Asian region. Our analysis of these whole genome sequences has provided novel insights into the genomic attributes that underlie the attenuated virulence of the *F. tularensis* subsp. *holarctica* and *F. tularensis* subsp. *mediasiatica* lineages, in comparison to *F. tularensis* subsp. *tularensis* strains. These subspecies are more closely related to each other phylogenetically than to their more distant *philomiragia* and *novicida* relatives, however, they inhabit geographically distinct regions. Although the origin of the Type B lineage is debatable, recent evidence suggests that *F. tularensis* subsp. *holarctica* originated in Asia, as evident from the phylogenetic basal positioning of the *F. tularensis* subsp. *holarctica* FSC022 *japonica* strain, and this clade diverged from the Type-B radiation lineage proposed to have originated in North America. Molecular approaches prior to the availability of whole genome sequence analysis has characterized the Type B-radiation lineage as a genetically homogeneous clade. These findings together with the wide distribution of Type B isolates across the Northern hemisphere has lead to speculations that the Type B-radiation group recently emerged through a genetic bottleneck, resulting in niche adaptation and attenuation of virulence. Consequently, increased survival in a range of vertebrate and invertebrate hosts likely facilitated the spread of Type B *Francisella* across the Northern hemisphere. The findings that Type B strains can survive in watercourses also reflects this broadening of the host range.

Here, we provide evidence suggesting that genome rearrangements and gene decay may have played a prominent role in the pathoadaptation of different *Francisella* subspecies, including the Type B-radiation lineage. Our findings therefore suggest that a greater genetic diversity exists between isolates of the Type B group than previously known. Since these strains are more recently diverged in comparison to other *Francisella* strains, our findings also suggest that perhaps this lineage is undergoing an evolutionary process of further diversification. Evidence of genomic rearrangements and diversity is predominant from our comparisons between the more ancestral FSC022 strain and all of the other Type B strains of the radiation lineage. Surprisingly, we also report similar evidence of significant rearrangements from comparisons specifically between the OSU18 and FSC257 strains of the Type B-radiation lineage. In addition, we report cases of pseudogenes that are specific to Type A.II and Type B strains isolated from the Russian and Sweden regions, which include the FSC257 strain.

Like the Type B lineage, *F. tularensis* subsp. *mediasiatica* has also previously been characterized as a subspecies with very little genetic diversity. In contrast, however, *F. tularensis* subsp. *mediasiatica* is geographically very restricted and isolates are rare which may limit our knowledge of the true genetic diversity of this lineage. Interestingly, despite the distinction of their environmental niches, the *F. tularensis* subsp. *holarctica* and *F. tularensis* subsp. *mediasiatica* lineages share a similar pathogenicity profile and we find that the genomes of these subspecies share intriguing genomic profiles with respect to key genes known to play a role in bacterial pathogenesis. Specifically, the *F. tularensis* subsp. *holarctica* and *F. tularensis* subsp. *mediasiatica* strains contain polymorphisms in a shared set of genes that contain known factors important for virulence and niche adaptation in other bacteria. These genes encode protein products that include important transcriptional regulators (ie. LysR family), structural components (ie. PilT), metabolic regulators (ie. MdaB), membrane proteins, and transporters of the Major facilitator superfamily. Also disrupted specifically in the *F. tularensis* subsp. *holarctica* and *F. tularensis* subsp. *mediasiatica* strains is a gene of unknown function that is a component of the *Francisella* pathogenicity island (FTT1348) and encodes a protein fusion product of two hypothetical proteins in *F. tularensis* subsp. *novicida*. We also find that a gene encoding a subunit of the OppA oligopeptide transporter is disrupted in all of the radial lineage Type B strains and the *F. tularensis* subsp. *mediasiatica* strain, but is intact in the more ancestral FSC022 strain. Also of note is the specific disruption of the *msrA2* gene in *F. tularensis* subsp. *mediasiatica* that encodes a peptide methionine sulfoxide reductase.

The collection of genes that we have identified as likely regulators of niche adaptation and virulence in *Francisella* also includes genes encoding factors exhibiting weak sequence similarity to known genes of the Type III and Type IV secretion systems. The biological significance of the sequence similarity of some *Francisella* loci to genes encoding effector and component proteins characteristic of either the T3SS or T4SS, respectively, is unclear. The absence of intact Type III or Type IV secretion systems might be indicative of evolutionary decay, or a mechanism of cross-talk between components of partial secretion systems that mediate Francisella virulence; An aspect which emphasizes the uniqueness of the mechanism of virulence in Francisella in comparison to other bacteria.

Our findings shed light on the evolutionary process of Francisella pathogenicity, and also provide broader insight into the general evolutionary process of bacterial pathoadaptation. A model of evolution of *Francisella* subspecies can be proposed from comparative analysis of genomic features of *F. tularensis* subspecies and what is known regarding mechanisms of pathoadaptation in recently obligate intracellular pathogens: As strains underwent geographical dispersion and adaptation to new niches, their genomes acquired more transposable elements and experienced higher frequencies of rearrangements. These events led to an increase of genomic polymorphisms that promoted functional acquisition required for environmental adaptation and virulence, as evident in the highly pathogenic *tularensis* subspecies. The high abundance of pseudogenes in more recently emerged subspecies is likely a reflection of the *Francisella* genomes in decay, especially in the strains that are human pathogens, where the more nutritionally rich environment in the host (e.g, rabbits and humans) made it unnecessary to maintain many of the genes required as a free-living organism. Interestingly, mechanisms of genomic pathoadaptation seems to have promoted a more benign pathogenic biology in the more recently emerged Type B *Francisella* strains, and attenuation in rarely human pathogenic *F. tularensis* subsp. *mediasiatica*.

## Materials and Methods

### Sequencing and Assembly

*F. tularensis* subsp. *holarctica* type B strain FSC257 (FSC257) was isolated from the tick Dermacentor pictus in 1949 from the area of Moscow, Russia. A different lineage of *holarctica* subspecies, strain FSC022, was isolated in Japan in 1950. The CDC standard for Type A strains, FSC033 (399), was isolated from a squirrel in Georgia, USA and the genomic DNA of this strain, as well as strains FSC257 and FSC022 were kindly provided by Mats Forsman of Swedish Defense Research Agency, Sweden.

Two antibiotic sensitive *F. tularensis* subsp. *novicida* strains, GA99-3548 and GA99-3549(F6168) [75], were isolated from human patient samples in Louisiana and California, respectively and the DNA sequenced in this project was provided by Scott Bearden at the CDC Fort Collins, Colorado, USA.

8× draft assemblies of 5 strains using 454 Technology and ABI Hybrid Assembly was done as described at http://www.broad.mit.edu/seq/msc/. The sequence coverage generated is shown in Table 1 and Arachne [76] was used for sequence assembly.

### Accession Numbers

The 8× draft assemblies for the 5 strains were deposited in GenBank and the accession numbers for these sequences, as well as the accession numbers for publicly available *Francisella* sequences also used in this study, are provided in Table 7.

**Table 7:** GenBank Accession Numbers for Francisella Strains Used in This Study.

GenBank accession numbers	Strain name (strains sequenced by the Broad in bold)
AJ749949	*F. tularensis* subspecies *tularensis* strain SCHU S4
***AAYE00000000***	***F. tularensis subspecies tularensis FSC033***
AM286280	*F. tularensis* subsp. *tularensis* FSC198
CP000608	*F. tularensis* subsp. *tularensis* WY96-3418
CP000437	*F. tularensis* subsp. *holarctica* OSU18
CP000803	*F. tularensis* subsp. *holarctica* FTA
***AAUD00000000***	***F. tularensis*** subsp. ***holarctica FSC257***
***AAYD00000000***	***F. tularensis*** subsp. ***holarctica FSC022***
NC_007880	*F. tularensis* subsp. *holarctica* LVS
AASP00000000	*F. tularensis* subsp. *holarctica* FSC200
CP000439	*F. tularensis* subsp. *novicida* U112
***ABAH00000000***	***F. tularensis*** subsp. ***novicida GA99-3548***
***AAYF00000000***	***F. tularensis*** subsp. ***novicida GA99-3549***
CP000937	*F. philomiragia* subsp. *philomiragia* ATCC 25017

Strain sequences available from the Microbial Genome Projects at BCM-HGSC: [86]
*Francisella tularensis* subsp. *tularensis* ATCC6223
*Francisella tularensis* subsp. *holarctica* KO971026
*Francisella tularensis* subsp. *holarctica* MI001730
*Francisella tularensis* subsp. *holarctica* OR96246
*Francisella tularensis* subsp. *holarctica* RC503

### Annotation and Analysis

The *Francisella* genomes were annotated as described on the Broad Institute *Francisella tularensis* group database: (http://www.broad.mit.edu/annotation/genome/Francisella_tularensis_group/MultiHome.html)

### Genomic Comparative Maps

Genomic Comparative Maps were constructed using CGview software [77] and scripts for mapping blast analysis provided courtesy of Paul Stothard and customized by M.Champion. Five sequenced draft genomes (*F. tularensis* subsp. *holarctica* FSC257 and FSC022; *F. tularensis* subsp. *tularensis* FSC033; *F. tularensis* subsp. *novicida* GA99-3548, and GA99-3549 strains) were aligned to the *F. tularensis* subsp. *holarctica* OSU18 reference sequence using the blastn program (minimum percent identity = 95 and expected threshold = 1e^-5^).

### Genome Alignments and Analysis of Predicted Genome Rearrangements

A comparison of genome rearrangement patterns between the more ancestral FSC022 Type B strain (reference) and the clonal strains of the *holarctica* subspecies was done using the alignment program, PatternHunter and for certain cases, visual comparisons were also done using Mauve [78]. PatternHunter was utilized at default settings; Except the maximum distance between spans for the spans to be merged on both the reference and the query sequence was set to 200 bp and the alignments were filtered for overlap percentages > = 90 [79]. Predicted rearrangements were identified from sequence alignments and breakpoint sites were further analyzed for proximal gene decay (see methods below for further description of how pseudogenes were identified). Specifically, total predicted ORF counts were binned according to distance (kb) from the predicted breakpoint. The number of pseudogenes in each bin was used to determine the percentage present. The statistical significance of pseudogene enrichment was determined using Fisher's Exact Test to derive p-values. PatternHunter whole genome alignments were also used to generate dotplots. For confirmation of select breakpoints in Mauve, a default LCB cutoff of 175, and filtering for blocks > =  to 10 kb.

### Phylogenetic Analysis

The evolutionary history of 20 *Francisella* strains was inferred from whole genome SNP data using the Maximum Parsimony method and MEGA4 software [80]. PatternHunter was utilized (as described above) to perform pairwise local alignments of 200 bp segments on a sliding window. This analysis compared sequence segments and did not require synteny, therefore rearrangements were not a factor in SNP discovery. Whole genome SNPs at least 20 bp apart and present in more than one *Francisella* genome were identified using custom scripts and clustalw alignments of these sequences were selected for further analysis using MEGA4 software. Specifically, the bootstrap consensus tree inferred from 500 replicates is taken to represent the evolutionary history of the taxa analyzed (Figure 2). Branches corresponding to partitions reproduced in less than 50% bootstrap replicates are collapsed. The percentage of replicate trees in which the associated taxa clustered together in the bootstrap test (500 replicates) are shown above the branches. The MP tree was obtained using the Close-Neighbor-Interchange algorithm with search level 3 in which the initial trees were obtained with the random addition of sequences (10 replicates). The tree is drawn to scale, with branch lengths calculated using the average pathway method and are in the units of the number of changes over the whole sequence. All positions containing gaps and missing data were eliminated from the dataset (Complete Deletion option). There were a total of 6180 positions in the final dataset, out of which 6164 were parsimony informative.

### Comparative Analysis of Gene Content

We performed an extensive comparative analysis of ORFs and whole genome sequences to identify genes unique to one or more subspecies, but absent in others. Briefly, we did an all versus all blast comparison (e = 10^−10^) of all CDS (including pseudogenes) from four completely sequences genomes: SCHU S4 (Type A1); WY96-3418 (Type A2), OSU18 (Type B) and U112 (spp. *novicida*), to identify an initial list of ORFs not found in ORFs from one or more of the other genomes. We then used this list of ORFs to search against all 20 genomes in order to evaluate conservation profiles using both nucleotide as well as protein sequence alignments (clustalw).

We used a similar approach to search for likely gene fusion/split events. Specifically, we first identified candidate gene pairs via an all versus all blast comparison (e = 10^−10^) of coding sequences. We used custom scripts to identify candidates and then reviewed these candidates in the context of genome alignments and targeted gene sequence alignment from all the available *Francisella* genome sequences. This allowed us to distinguish real gene fusion/split event from artifacts due to annotation or sequencing errors.

We assigned all protein coding genes (including pseudogenes) to KEGG pathways using KAAS [81]. Additional information about transporters were added from [36], and from membrane transportDB and TransAAP analysis [82],[83]. Members of the bacterial two component systems were identified using PFAM domains for the response regulators (PF00072) and histidine kinase (PF00512, PF07536, PF07568 and PF0773), similar to the approach used by Kiil et al. [84].

#### Search for similarity to proteins in the T3SS and T4SS pathways

We used *P. aeruginosa* T3SS component protein sequences and a repertoire of effector protein sequences as queries to search a translated nucleotide *Francisella* database with the TBLASTN algorithm [85] (BLOSUM62 matrix, default parameters with the exception of setting the expected threshold to e<1^e-5^) similar to the approach of Tobe, *et. al.* [48]. The same approach, using *Agrobacterium tumefaciens* C58 T4SS protein sequences as queries was also done [52],[53]. In addition, we also used the *Francisella* nucleotide sequences as queries to search the KEGG peptide database using blastx (BLOSUM62 matrix, default parameters with the exception of setting the expected threshold to e<1^e-5^). Hits were filtered based on the expectation threshold, however in general, those identified also exhibit a <40% identity to the query and therefore are defined as ‘weakly similar’.

## Supporting Information

Figure S1 Whole genome sequence alignments and dotplot comparisons between the *F. tularensis* subsp. *mediasiatica* FSC147 strain and other subspecies strains. FSC147 is the reference genome (X axis) in all dotplot comparisons (A–C). (A) *F. tularensis* subsp. *mediasiatica* FSC147 and *F. tularensis* subsp. *novicida* GA99-3548, (B) *F. tularensis* subsp. *mediasiatica* FSC147 and *F. tularensis* subsp. *holarctica* FSC022, (C) *F. tularensis* subsp. *mediasiatica* FSC147 and *F. tularensis* subsp. *tularensis* SCHU S4. Alignments were filtered for overlap percentages greater than or equal to 90%. Numerous rearrangements are evident from the dotplot comparisons.
(0.56 MB TIF)

Table S1 Subspecies specific disruption of genes encoding proteins of major secretory pathways, membrane proteins and components of known metabolic pathways.
(0.40 MB DOC)

Table S2 Summary of genes identified as candidates for mediating pathogenicity in *F. tularensis* subsp. *tularensis* (Type A), *F. tularensis* subsp. *holarctica* (Type B), and *F. tularensis* subsp. *novicida* by previous studies.
(0.15 MB DOC)
