# Supplementary figures and images for: Comparative Genomic Characterization of Francisella tularensis Strains Belonging to Low and High Virulence Subspecies
Source: PLoS Pathog. 2009 May 29;5(5):e1000459. doi: 10.1371/journal.ppat.1000459 (PMC2682660; doi:10.1371/journal.ppat.1000459)

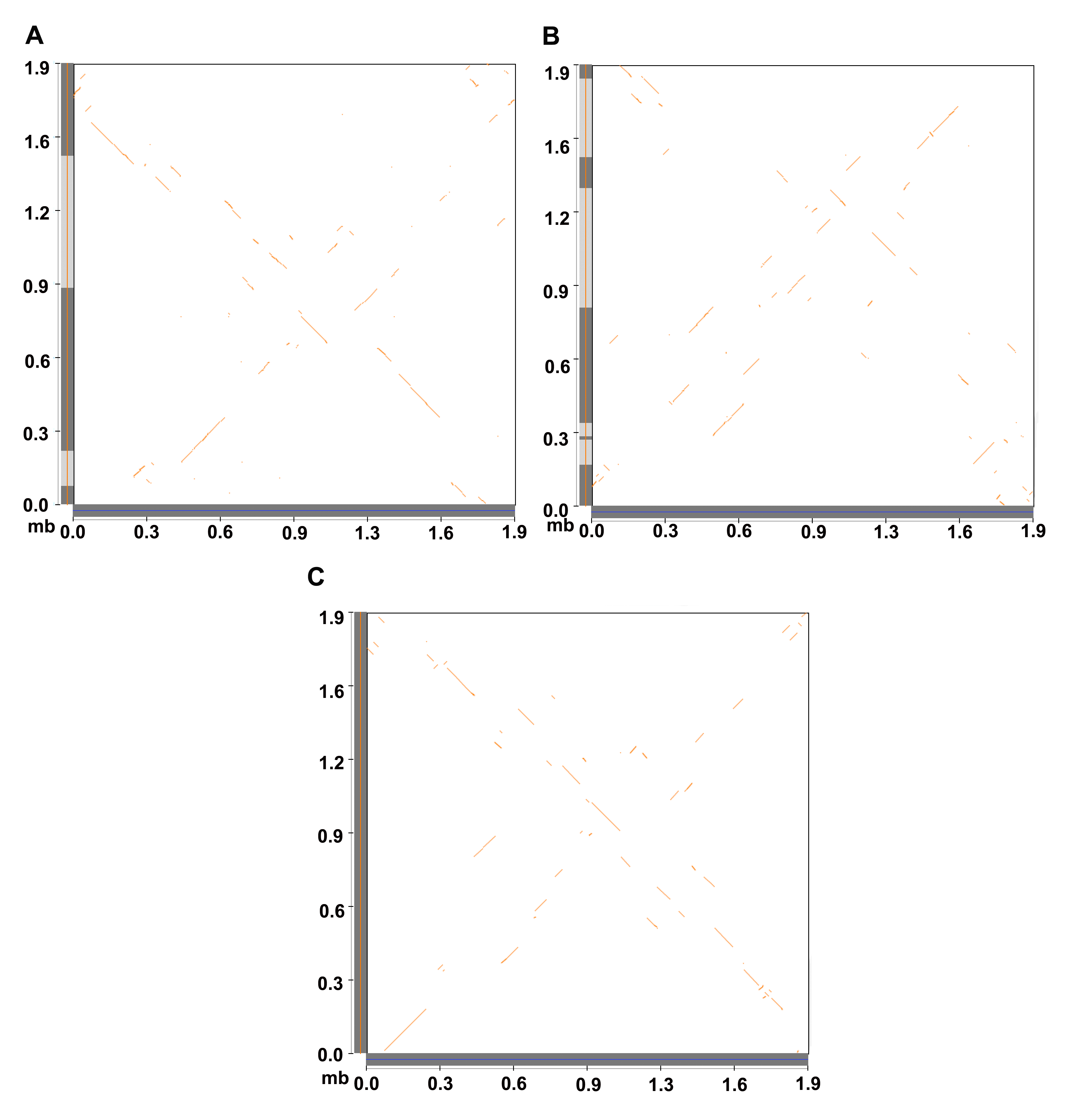

Supplement: Figure S1 — Whole genome sequence alignments and dotplot comparisons between the F. tularensis subsp. mediasiatica FSC147 strain and other subspecies strains. FSC147 is the reference genome (X axis) in all dotplot comparisons (A–C). (A) F. tularensis subsp. mediasiatica FSC147 and F. tularensis subsp. novicida GA99-3548, (B) F. tularensis subsp. mediasiatica FSC147 and F. tularensis subsp. holarctica FSC022, (C) F. tularensis subsp. mediasiatica FSC147 and F. tularensis subsp. tularensis SCHU S4. Alignments were filtered for overlap percentages greater than or equal to 90%. Numerous rearrangements are evident from the dotplot comparisons. (0.56 MB TIF) [file ppat.1000459.s001.tif]
