# Supplementary material for: Comparative Genomic Characterization of Francisella tularensis Strains Belonging to Low and High Virulence Subspecies
Source: PLoS Pathog. 2009 May 29;5(5):e1000459. doi: 10.1371/journal.ppat.1000459 (PMC2682660; doi:10.1371/journal.ppat.1000459)
Supplement: Table S1 — Subspecies specific disruption of genes encoding proteins of major secretory pathways, membrane proteins and components of known metabolic pathways. (0.40 MB DOC) [file ppat.1000459.s002.doc]

**Supplemental Table 1a *Francisella* Metabolic Pathways** [50]

| **Amino Acid metabolism** | |
| --- | --- |
| KO# | Description |
| **Disrupted or absent in *F. tularensis* subsp. *tularensis* (Type A), *F. tularensis* subsp. *holarctica* (Type B), and *F. tularensis* subsp. *mediasiatica*** | |
| ko00251 | Glu metabolism; NE |
| ko00251 | Gln metabolism; NE |
| ko00252 | Ala metabolism; NE |
| ko00252 | Asp metabolism |
| ko00252 | Asn metabolism; NE |
| ko00272 | Cys metabolism |
| ko00300 | Lys metabolism |
| ko00330 | Pro metabolism |
| ko00330 | Arg metabolism |
| ko00340 | His metabolism |
| ko00400 | Phe metabolism; NE |
| ko00400 | Trp metabolism; NE |
| ko00290 | Val metabolism |
| ko00290 | Ile metabolism |
| ko00290 | Leu metabolism |
| ko00400 | Tyr metabolism |
|  | |
| **Intact only in *F. tularensis* subsp. *novicida* strains** | |
| k000260 | Gly metabolism; NE |
| k000260 | Ser metabolism |
| k000260 | Thr metabolism |
| ko00271 | Met metabolism |
|  | |
| **Other Metabolic Pathways** | |
| **Disrupted or absent in *F. tularensis* subsp. *tularensis* (Type A), *F. tularensis* subsp. *holarctica* (Type B), and *F. tularensis* subsp. *mediasiatica*** | |
| ko00230 | Purine metabolism |
| ko00030 | Enzyme in pentose phosphate pathway (2.7.1.5) |
| ko00010 | Glycolysis / Gluconeogenesis |
|  |  |
| **Intact in all *Francisella* subspecies** | |
| ko00240 | Pyrimidine metabolism |
|  | |
| **Intact only in *F. philomiragia* subsp. *philomiragia* strains** | |
| ko00220 | Urea cycle and metabolism of amino groups |

NE: nonessential amino acids

**Supplemental Table 1b *Francisella*** genes encoding transporters

| **Disrupted or absent in *F. tularensis* subsp. *mediasiatica* FSC147 only:** | |
| --- | --- |
| FTT0157c | licB-like transmembrane protein |
| FTT0442c | major facilitator superfamily (MFS) transporter |
| FTT0670c | hypothetical membrane protein |
| FTT0686c | proton-dependent oligopeptide transport (POT) family protein |
| FTT0728 | ABC transporter, ATP-binding protein ybhF |
| FTT0804 | major facilitator superfamily (MFS) transporter |
| FTT1090 | hypothetical membrane protein |
| FTT1124 | D-methionine transport protein, ABC transporter,ATP-binding subunit |
| FTT1277c | sodium-solute symporter |
| FTT1339c | sulfate permease |
| FTT1510c | aromatic amino acid transporter, HAAAP family |
|  |  |
| **Other disrupted or absent genes encoding transporters in various subspecies:** | |
| FTT0003c | major facilitator superfamily (MFS) transporter |
| FTT0006 | major facilitator superfamily (MFS) transporter |
| FTT0017 | ABC transporter |
| FTT0122 | oppA, oligopeptide transporter, subunit A |
| FTT0123 | oppB, oligopeptide transporter, subunit B |
| FTT0124 | oppC, oligopeptide transporter, subunit C |
| FTT0125 | oppD, oligopeptide transporter, subunit D |
| FTT0126 | oppF, oligopeptide transporter, subunit F |
| FTT0129 | major facilitator superfamily (MFS) transporter |
| FTT0176c | ABC transporter for organic cation/carnitine (OCTN) |
| FTT0201 | aromatic amino acid transporter HAAAP family |
| FTT0210c | major facilitator superfamily (MFS) transporter |
| FTT0225c | major facilitator superfamily (MFS) transporter |
| FTT0246c | major facilitator superfamily (MFS) transporter |
| FTT0275c | major facilitator superfamily (MFS) transporter DHA1 |
| FTT0276c | ABC transporter |
| FTT0310 | amino acid permease |
| FTT0361c | amino acid transporter |
| FTT0375 | The p-aminobenzoyl-glutamate transporter family |
| FTT0445 | ABC transporter |
| FTT0446 | proton-dependent oligopeptide transport (POT) family protein |
| FTT0475 | mechanosensitive ion channel protein msc |
| FTT0487 | major facilitator superfamily (MFS) transporter |
| FTT0488c | major facilitator superfamily (MFS) transporter |
| FTT0493 | major facilitator superfamily (MFS) transporter |
| FTT0498c | proton-dependent oligopeptide transport (POT) family protein |
| FTT0526 | ABC transporter |
| FTT0527 | ABC transporter |
| FTT0528 | ABC transporter |
| FTT0531 | ABC transporter, ATP-binding protein |
| FTT0567c | proton-dependent oligopeptide transport (POT) family protein |
| FTT0572 | proton-dependent oligopeptide transport (POT) family protein |
| FTT0574 | amino acid permease |
| FTT0600 | major facilitator superfamily (MFS) transporter |
| FTT0604 | The Monovalent Cation:Proton Antiporter-1 (CPA1) Family |
| FTT0651 | proton-dependent oligopeptide transport (POT) family protein |
| FTT0657 | major facilitator superfamily (MFS) transporter |
| FTT0671 | major facilitator superfamily (MFS) transporter |
| FTT0672c | major facilitator superfamily (MFS) transporter |
| FTT0685c | ABC transporter, Voltage-Gated Ion Channel (VIC) superfamily |
| FTT0727 | ABC transporter (Exporters:DRI/YHIH) |
| FTT0729 | ABC transporter, membrane protein (Exporters:DRI/YHIH) |
| FTT0740c | organic solvent tolerence protein ostA2 |
| FTT0744c | hypothetical protein |
| FTT0745c | hypothetical protein |
| FTT0746c | hypothetical protein |
| FTT0775c | major facilitator superfamily (MFS) transport protein bcr2 |
| FTT0829c | aspartate:alanine antiporter |
| FTT0849 | bile acid symporter |
| FTT0853 | arsenite permease arsB |
| FTT0866c | cadmium-transporting ATPase |
| FTT0931 | major facilitator superfamily (MFS) transporter |
| FTT0947c | major facilitator superfamily (MFS) transporter |
| FTT0992 | mechanosensitive ion channel protein msc |
| FTT0995 | major facilitator superfamily (MFS) transporter |
| FTT0999c | metal transporter family protein ZIP family |
| FTT1005c | proton-dependent oligopeptide transport (POT) family protein |
| FTT1006 | hypothetical membrane protein |
| FTT1020c | amino acid permease |
| FTT1032 | hypothetical membrane protein |
| FTT1104 | major facilitator superfamily (MFS) transporter bcr1 |
| FTT1107c | betaine/carnitine/choline transporter (BCCT) family protein betT |
| FTT1118c | hypothetical membrane protein |
| FTT1126 | aromatic amino acid transporter HAAAP family |
| FTT1189c | major facilitator superfamily (MFS) transporter |
| FTT1233c | proton-dependent oligopeptide transport (POT) family protein yjdL |
| FTT1311 | major facilitator superfamily (MFS) transporter yieO |
| FTT1362 | major facilitator superfamily (MFS) transporter |
| FTT1380 | major facilitator superfamily (MFS) transporter |
| FTT1399 | hypothetical membrane protein |
| FTT1440c | major facilitator superfamily (MFS) transporter |
| FTT1453c | O-antigen flippase wzx |
| FTT1513 | amino acid permease |
| FTT1533c | major facilitator superfamily (MFS) transporter |
| FTT1618 | major facilitator superfamily (MFS) transporter |
| FTT1645 | major facilitator superfamily (MFS) transporter |
| FTT1670c | Na+:H+ antiporter |
| FTT1717 | major facilitator superfamily (MFS) transporter |
| FTT1729c | ABC transporter, ATP-binding protein |
| FTT1738c | potassium-transporting ATPase B chain kdpB |
| FTT1739c | potassium-transporting ATPase A chain kdpA |
| FTT1743 | Na+:H+ antiporter |
| FTT1757c | major facilitator superfamily (MFS) transporter |
| FTT1775c | the chloride channel family transporter |
| FTT1787c | transporter, lysE family |
| FTN_0004 | aspartate/glutamate transporter |
| FTN_0005 | divalent inorganic cation transporter corA |
| FTN_0008 | 10 TMS drug/metabolite exporter protein |
| FTN_0799 | membrane transporter of cations and cationic drugs, multidrug resistance protein smr/emrE |
| FTN_0800 | haloacid dehalogenase-like hydrolase family protein ACR3 |
| FTN_0932 | ABC transporter, ATP-binding protein |
| FTN_1079 | major facilitator superfamily (MFS) transporter; sugar porter (SP) family |
| FTN_1420 | O-antigen flippase wzx |

**Supplemental Table 1c *Francisella* genes encoding Secreted Proteins**

| **Inactive genes encoding secreted proteins in *F. tularensis* subsp. *mediasiatica* FSC147 only:** | |
| --- | --- |
| FTT0013c | hypothetical lipoprotein |
| FTT0014c | hypothetical protein |
| FTT0083 | hypothetical membrane protein |
| FTT0538c | hypothetical lipoprotein |
| FTT0784 | hypothetical protein |
| FTT1506 | hypothetical protein |
| FTT1550 | hypothetical protein |
|  |  |
| **Other disrupted or inactive genes encoding secreted proteins:** | |
| FTT0263 | hypothetical membrane protein |
| FTT0308 | hypothetical protein |
| FTT0465 | hypothetical protein |
| FTT0485 | hypothetical protein |
| FTT0543 | hypothetical protein |
| FTT0546 | hypothetical protein |
| FTT0613c | hypothetical protein |
| FTT0620 | HAD superfamily protein |
| FTT0678c | hypothetical lipoprotein |
| FTT0715 | chitinase family 18 protein |
| FTT0741c | hypothetical protein |
| FTT0747c | hypothetical protein |
| FTT0749c | hypothetical protein |
| FTT0768c | hypothetical protein |
| FTT0816c | chitin binding protein |
| FTT0826c | hypothetical lipoprotein |
| FTT0852 | hypothetical protein |
| FTT0869 | hypothetical protein |
| FTT0880 | hypothetical membrane protein |
| FTT0887c | hypothetical protein |
| FTT0902 | hypothetical protein |
| FTT0949c | hypothetical membrane protein |
| FTT0975 | hypothetical protein |
| FTT0987 | hypothetical protein |
| FTT0989 | hypothetical protein |
| FTT1008c | hypothetical protein |
| FTT1012 | hypothetical protein |
| FTT1082 | ATP-dependent helicase HEPA |
| FTT1109 | choloylglycine hydrolase family protein |
| FTT1153c | hypothetical protein |
| FTT1234 | choloylglycine hydrolase family protein |
| FTT1242 | hypothetical protein |
| FTT1286 | pseudogene, choloylglycine hydrolase family protein |
| FTT1307c | hypothetical protein |
| FTT1492c | hypothetical protein |
| FTT1588c | hypothetical protein |
| FTT1591 | lipoprotein |
| FTT1593c | hypothetical protein |
| FTT1598 | hypothetical membrane protein |
| FTT1621c | hypothetical membrane protein |
| FTT1625c | hypothetical protein |
| FTT1682 | hypothetical protein |
| FTT1776c | hypothetical membrane protein |
| FTT1789 | hypothetical protein |

**Table 1d *Francisella*** genes encoding membrane proteins

| **Inactive genes encoding membrane proteins in *F. tularensis* subsp. *mediasiatica* FSC147 only:** | |
| --- | --- |
| FTT0755 | hypothetical membrane protein |
| FTT0787 | DoxD-like family protein |
| FTT0903 | hypothetical protein |
| FTT1016c | GDSL-like lipase/acylhydrolase family protein |
| FTT1246 | hypothetical protein |
| FTT1423c | hypothetical membrane protein |
| FTT1622c | hypothetical membrane protein |
|  |  |
| **Other disrupted or absent genes encoding membrane proteins:** | |
| FTT0018 | secretion protein |
| FTT0092c | pseudogene of appC, cytochrome oxidase bd-II, subunit I |
| FTT0159c | hypothetical membrane protein (truncation in A)  *pseudogene in Russia/Sweden Type B strains |
| FTT0172 | hypothetical membrane protein |
| FTT0179 | pseudogene of rec2, DNA internalization-related competence protein |
| FTT0194c | hypothetical protein |
| FTT0214 | transport protein |
| FTT0218c | pseudogene, cytochrome b561 family protein |
| FTT0260 | CrcB family protein |
| FTT0262 | hypothetical lipoprotein |
| FTT0272 | hypothetical membrane protein |
| FTT0302 | hypothetical membrane protein |
| FTT0376c | hypothetical membrane protein |
| FTT0427 | pseudogene of thrB, homoserine kinase |
| FTT0441c | pseudogene, NADH dehydrogenase subunit |
| FTT0447c | hypothetical protein |
| FTT0539c | pseudogene, thiF family protein |
| FTT0734 | hypothetical membrane protein |
| FTT0742 | hypothetical lipoprotein |
| FTT0754c | hypothetical membrane protein |
| FTT0851 | hypothetical protein |
| FTT0861c | Type IV pili fiber building block protein |
| FTT0921 | hypothetical membrane protein |
| FTT0965c | hypothetical protein |
| FTT0967c | hypothetical membrane protein |
| FTT0983 | hypothetical membrane protein |
| FTT1009 | hypothetical membrane protein |
| FTT1011 | hypothetical protein |
| FTT1047c | hypothetical protein |
| FTT1048c | hypothetical protein |
| FTT1049c | pseudogene of cysC, adenylylsulfate kinase |
| FTT1080c | hypothetical membrane protein |
| FTT1163c | hypothetical membrane protein |
| FTT1172c | cold shock protein (DNA-binding) csp |
| FTT1175c | hypothetical membrane protein |
| FTT1178c | hypothetical membrane protein |
| FTT1188 | hypothetical membrane protein |
| FTT1261c | pseudogene, flavodoxin family protein |
| FTT1381 | hypothetical membrane protein |
| FTT1385c | hypothetical protein |
| FTT1426c | hypothetical membrane protein |
| FTT1437c | hypothetical membrane protein |
| FTT1505c | hypothetical membrane protein |
| FTT1514c | hypothetical membrane protein |
| FTT1552 | Delta 9 acyl-lipid fatty acid desaturase ole1 |
| FTT1580c | hypothetical protein |
| FTT1624c | hypothetical protein |
| FTT1626c | hypothetical membrane protein |
| FTT1627c | hypothetical protein |
| FTT1628c | metal ion transporter |
| FTT1658c | hypothetical protein |
| FTT1770 | hypothetical membrane protein |
| FTT1784c | hypothetical protein |

**Supplemental Table 1e Other genes disrupted or absent in *Francisella* subspecies**

(Excluding genes encoding transporters, secreted proteins and membrane proteins)

| **Inactivated in *F. tularensis* subsp. *mediasiatica* FSC147 only:** | |
| --- | --- |
| FTT0005 | succinate-semialdehyde dehydrogenase, fragment |
| FTT0136 | helicase |
| FTT0155 | oxidoreductase iron/ascorbate family protein |
| FTT0185 | D-alanine-D-alanine ligase B ddlB |
| FTT0195c | L-glutaminase |
| FTT0251 | branched-chain amino acid aminotransferase protein (class IV) ilvE |
| FTT0305 | MutT/nudix family protein |
| FTT0395 | hypothetical protein |
| FTT0403 | peptide deformylase def1 |
| FTT0406 | lysine decarboxylase, inducable cadA |
| FTT0425c | aspartate-semialdehyde dehydrogenase asd |
| FTT0428 | threonine synthase thrC |
| FTT0602c | hypothetical protein |
| FTT0814c | hypothetical protein |
| FTT0932 | ROK family protein |
| FTT0980 | hypothetical protein |
| FTT1081c | hypothetical protein |
| FTT1086c | hypothetical protein |
| FTT1106 | tryptophan-rich sensory protein tspO |
| FTT1131 | pseudogene, molybdopterin binding family protein |
| FTT1187 | hypothetical protein |
| FTT1212c | lactoylglutathione lyase gloA |
| FTT1559c | pyrroline-5-carboxylate reductase proC |
| FTT1581c | endonuclease |
| FTT1614c | hypothetical protein |
| FTT1677c | tRNA (5-methylaminomethyl-2-thiouridylate)-methyltransferase trmU |
| FTT1774c | pseudogene, carboxypeptidase |
| FTT1785c | hypothetical protein |
| FTT1798c | hypothetical protein |
|  |  |
| **Other disrupted or absent genes:** | |
| FTT0010 | modification methylase, fragment |
| FTT0024c | hypothetical protein |
| FTT0069c | hypothetical protein |
| FTT0082 | pseudogene, transcriptional regulator |
| FTT0089c | hypothetical protein |
| FTT0096 | hypothetical protein |
| FTT0170c | hypothetical protein |
| FTT0173 | hypothetical protein |
| FTT0200 | hypothetical protein |
| FTT0206c | pseudogene, dienelactone hydrolase family protein |
| FTT0217 | hypothetical protein |
| FTT0222c | hydrolase subunit ybgK |
| FTT0224c | hypothetical protein |
| FTT0227c | hypothetical protein |
| FTT0241c | hypothetical protein |
| FTT0254c | hypothetical protein |
| FTT0248 | hypothetical protein |
| FTT0255c | hypothetical protein |
| FTT0267 | hypothetical protein |
| FTT0311c | hypothetical protein |
| FTT0358 | hypothetical protein |
| FTT0401 | hypothetical protein |
| FTT0433 | hypothetical protein |
| FTT0434 | hypothetical protein |
| FTT0435 | carbon-nitrogen hydrolase family protein |
| FTT0490c | phospholipase D family protein. |
| FTT0492c | transcriptional regulator, lysR family |
| FTT0497c | pseudogene, asparaginase 2 family protein |
| FTT0499 | hypothetical protein |
| FTT0516 | pseudogene, oxidoreductase |
| FTT0525 | hypothetical protein |
| FTT0547 | hypothetical protein |
| FTT0551 | hypothetical protein |
| FTT0566 | hypothetical protein |
| FTT0584 | hypothetical protein |
| FTT0585 | hypothetical protein |
| FTT0586 | hypothetical protein |
| FTT0605c | hypothetical protein |
| FTT0606c | hypothetical protein |
| FTT0637 | hypothetical protein |
| FTT0638 | hypothetical protein |
| FTT0639 | hypothetical protein |
| FTT0662c | hypothetical protein |
| FTT0666c | methylpurine-DNA glycosylase family protein |
| FTT0717 | pseudogene of purU, formyltetrahydrofolate deformylase |
| FTT0735 | pseudogene, mandelate racemase/muconate lactonizing enzyme |
| FTT0770 | pseudogene of tag, DNA-3-methyladenine glycosylase I |
| FTT0778 | hypothetical protein |
| FTT0828c | pseudogene, L-aspartate-beta-decarboxylase |
| FTT0830c | pseudogene, Smf protein DNA processing chain A |
| FTT0847 | hypothetical protein |
| FTT0929c | pseudogene, D-beta-hydroxybutyrate dehydrogenase |
| FTT0930c | pseudogene, acetoacetate decarboxylase |
| FTT0933 | pseudogene, bifunctional protein birA |
| FTT0948c | aldo/keto reductase |
| FTT0950c | hypothetical protein |
| FTT0957c | hypothetical protein |
| FTT0960 | hypothetical protein |
| FTT0962 | ThiJ/PfpI family protein |
| FTT0974 | pseudogene, amidinotransferase family protein |
| FTT0984c | mutator protein mutT |
| FTT0988 | hypothetical protein |
| FTT0996 | pseudogene, cardiolipin synthetase |
| FTT1033 | pseudogene of yihQ, glycosyl hydrolases family 31 protein |
| FTT1034c | NADH dehydrogenase ndh |
| FTT1052c | hypothetical protein |
| FTT1071c | hypothetical protein |
| FTT1073c | hypothetical protein |
| FTT1076 | transcription regulator hipA |
| FTT1077c | hypothetical protein |
| FTT1091 | isochorismatase hydrolase family protein |
| FTT1098c | pseudogene, uvrD/REP helicase family protein |
| FTT1101 | pseudogene of yjeS, 4Fe-4S ferredoxin |
| FTT1135c | hypothetical protein |
| FTT1139 | pseudogene of phrB, deoxyribodipyrimidine photolyase |
| FTT1144 | pseudogene, short-chain dehydrogenase/reductase (SDR) family protein |
| FTT1146c | pseudogene of galM, aldose 1-epimerase |
| FTT1162c | pseudogene, FAD-binding family protein |
| FTT1176c | pseudogene of hsdR1, Type I restriction enzyme subunit R |
| FTT1177c | hypothetical protein |
| FTT1180 | hypothetical protein |
| FTT1192c | hypothetical protein |
| FTT1209c | pseudo gene of pepO, metallopeptidase family M13 protein |
| FTT1211c | hypothetical protein |
| FTT1289 | hypothetical protein |
| FTT1301c | pseudogene, purine/pyrimidine phosphoribosyl transferase |
| FTT1308c | hypothetical protein |
| FTT1343c | hypothetical protein |
| FTT1361c | hypothetical protein |
| FTT1364 | pseudogene of treA, trehalase |
| FTT1414 | hypothetical protein |
| FTT1415 | hypothetical protein |
| FTT1425c | NADH oxidase naoX |
| FTT1430c | pseudogene, methyltransferase |
| FTT1480c | hypothetical protein |
| FTT1491c | hypothetical protein |
| FTT1501 | hypothetical protein |
| FTT1519 | pseudogene, outer membrane lipoprotein |
| FTT1536c | hypothetical protein |
| FTT1545 | hypothetical protein |
| FTT1546 | hypothetical protein |
| FTT1547 | pseudogene, cyclopropane-fatty-acid-phospholipid synthase |
| FTT1553c | ribonuclease R rnr |
| FTT1558c | hypothetical protein |
| FTT1565c | glycosyl hydrolase, family 3 |
| FTT1579c | Type III restriction enzyme |
| FTT1582c | pseudogene, Type III restriction-modification system |
| FTT1594 | transcriptional regulator lysR family |
| FTT1597 | hypothetical protein |
| FTT1656c | hypothetical protein |
| FTT1659 | hypothetical protein |
| FTT1662c | pseudogene, acid phosphatase |
| FTT1684 | transcription regulator |
| FTT1691 | hypothetical protein |
| FTT1698c | formate dehydrogenase fdh fragment |
| FTT1716c | hypothetical protein |
| FTT1719c | modification methylase fragment |
| FTT1733 | hypothetical protein |
| FTT1741c | formate dehydrogenase fdh fragment |
| FTT1755 | succinate-semialdehyde dehydrogenase, fragment |
| FTT1759c | pseudogene, oxidase-like protein |
| FTT1771 | hypothetical protein |
| FTT1779 | hypothetical protein |
| FTT1788 | pseudogene of iciA, chromosome initiation inhibitor |

**Supplemental Table 1f *Francisella* Signaling and Secretion Systems**

|  |  |
| --- | --- |
| **Intact genes of Two Component Systems in *Francisella*** | |
| FTT0094c | sensor histidine kinase qseC |
| FTT1557c | two-component response regulator pmrA |
| FTT1737c | potassium-transporting ATPase C chain kdpC |
| FTT1740c | potassium-transporting ATPase F chain kdpF |
|  | |
| **Disrupted Two Component Systems in *Francisella*** | |
| FTT1543 | two component response regulator vicR |
| FTT1544 | two-component sensor histidine kinase vicK |
| FTT1739c | potassium-transporting ATPase A chain kdpA |
| FTT1738c | potassium-transporting ATPase B chain kdpB |
| FTT1736c | two component sensor protein kdpD |
| FTT1735c | transcriptional regulatory protein kdpE |
| Fphi_0794 | signal transduction histidine kinase ntrY |
| Fphi_0797 | signal transduction response regulator receiver protein ntrX |
|  |  |
| **Intact potential substrates for TAT secretion pathway** | |
| FTT0702 | uridine kinase udk |
| FTT0819 | 50S ribosomal protein L35 rmpI |
| FTT0955c | glutathione reductase Gor |
| FTT0979c | Aminonacid permease |
| FTT1606 | septum sitendetermining protein minD |
| FTT1633c | amino acid transporter |
| FTT1640 | osmoprotectant transporter proP |
|  |  |
| **Disrupted potential substrates for TAT secretion pathway** | |
| FTT0361c | amino acid transporter |
| FTT0723c | short-chain dehydrogenase/reductase family protein |
| FTT0775c | major facilitator superfamily (MFS) transport protein bcr2 |
| FTT1032 | conserved hypothetical membrane protein |
| FTT1510c | aromatic amino acid transporter, HAAAP family |
|  |  |
| **Disrupted genes in predicted secretion systems** | |
| **T1SS** | **Description** |
| FTT0740c | organic solvent tolerence protein ostA2 |
| **T2SS** |  |
| FTT0088 | Type IV pili nucleotide-binding protein pilT |
| FTT0683c | Type IV pili leader peptidase and methylase pilD |
| FTT0861c | Type IV pili fiber block protein |
| FTT0888c | Type IV pili fiber building block protein |
| FTT0889c | Type IV pili fiber building block protein |
| FTT0890c | Type IV pili fiber building block protein |
| **T6SS** | ***Francisella* Pathogenicity Island** |
| FTT1348 | hypothetical protein |
| FTT1360c | hypothetical protein pdpD |
| **Intact genes in predicted secretion systems** | |
| **T6SS** | ***Francisella* Pathogenicity Island** |
| FTT1344 | hypothetical protein pdpA |
| FTT1345 | hypothetical protein pdpB |
| FTT1346 | hypothetical protein |
| FTT1347 | hypothetical protein |
| FTT1349 | hypothetical protein |
| FTT1350 | hypothetical protein |
| FTT1351 | hypothetical protein |
| FTT1352 | hypothetical protein |
| FTT1353 | hypothetical protein |
| FTT1354 | hypothetical protein pdpC |
| FTT1355 | hypothetical protein |
| FTT1356c | intracellular growth locus, subunit D iglD |
| FTT1357c | intracellular growth locus, subunit C iglC |
| FTT1358c | intracellular growth locus, subunit B iglB |
| FTT1359c | intracellular growth locus, subunit A iglA |

**Supplemental Table 1g Gene Fusions in *Francisella* Genomes**

| SchuS4 | OSU18/  WY96-3418 | Description (Schu S4 Ref) |
| --- | --- | --- |
| FTT0125 | FTH_1592 | oppD, peptide/opine/nickel uptake transporter (PepT) family protein |
| FTT0126 |  | oppF, peptide/opine/nickel uptake transporter (PepT) family protein |
| FTT0520 | FTH_0955 | hypothetical protein |
| FTT0521 |  | hypothetical protein |
| FTT0522 |  | Type I restriction-modification system, M subunit |
| FTT0523 |  | Type I restriction-modification system, DNA specificity subunit |
| FTT0843 | FTH_0335 | tRNA-methylthiotransferase MiaB |
| FTT0844 |  | short-chain alcohol dehydrogenase |
| FTT1194c | FTW_1242 | hypothetical protein |
| FTT1195c |  | hypothetical protein |
| FTT1437c | FTH_0624 | hypothetical protein |
| FTT1438c |  | hypothetical protein |

**Supplemental Table 1h Gene Splits in *Francisella*** Genomes

| **U112 ref** | **SCHU_S4/**  **OSU18 ref** | **Note** |
| --- | --- | --- |
| FTN_0067 | FTT0248  FTT0227c | hypothetical protein |
| FTN_0086 | FTT0225c  FTT0246c | metabolite:H+ symporter (MHS) family protein |
| FTN_0094 | FTT0217  FTT1691 | transcriptional regulator lysR family |
| FTN_0115 | FTT1670c  FTT1743 | Na+/H+ antiporter |
| FTN_0127 | FTT0005  FTT1755 | succinate semialdehyde dehydrogenase (NAD(P)+ dependent) gabD |
| FTN_0148 | FTT0172  FTT0272 | hypothetical membrane protein |
| FTN_0176 | FTH_1817 FTH_1848 | serine permease |
| FTN_0465 | FTH_0393 FTH_0434 | hypothetical protein sua5/ycio/yrdC family |
| FTN_0531 | FTH_0506 FTH_1572 | tRNA/rRNA methyltransferase yjfH |
| FTN_0681 | FTT0778  FTT1480c | acid phosphatase/phosphotransferase |
| FTN_1719 | FTT1698c FTT1741c | formate dehydrogenase fdh |
| FTH_1788 | FTT0010  FTT1719c | DNA modification methyltransferase *pseudogene in OSU18 pseudogene |
