# Supplementary material for: Comparative Genomic Characterization of Francisella tularensis Strains Belonging to Low and High Virulence Subspecies
Source: PLoS Pathog. 2009 May 29;5(5):e1000459. doi: 10.1371/journal.ppat.1000459 (PMC2682660; doi:10.1371/journal.ppat.1000459)
Supplement: Table S2 — Summary of genes identified as candidates for mediating pathogenicity in F. tularensis subsp. tularensis (Type A), F. tularensis subsp. holarctica (Type B), and F. tularensis subsp. novicida by previous studies. (0.15 MB DOC) [file ppat.1000459.s003.doc]

**Supplemental Table 2 Summary of genes identified as candidates for mediating pathogenicity in *F. tularensis* subsp. *tularensis* (Type A), *F. tularensis* subsp. *holarctica* (Type B), and *F. tularensis* subsp. *novicida* by previous studies [1; 3; 7; 8; 6; 2; 4]:**

| FTN_1756 | bacterioferritin comigratory protein bcp |
| --- | --- |
| FTT0046 | magnesium chelatase family protein comM |
| FTT0088 | Type IV pili nucleotide-binding protein pilT |
| FTT0122 | oppA, oligopeptide transporter, subunit A |
| FTT0125 | oppD, oligopeptide transporter, subunit D |
| FTT0185 | D-alanine-D-alanine ligase B ddlB |
| FTT0218c | pseudogene, cytochrome b561 family protein |
| FTT0361c | amino acid transporter |
| FTT0376c | hypothetical membrane protein |
| FTT0393 | methionine aminopeptidase map |
| FTT0395 | hypothetical protein |
| FTT0406 | lysine decarboxylase, inducable cadA |
| FTT0434 | hypothetical protein |
| FTT0435 | carbon-nitrogen hydrolase family protein |
| FTT0446 | proton-dependent oligopeptide transport (POT) |
| FTT0458 | stringent starvation protein A regulator of |
| FTT0487 | major facilitator superfamily (MFS) transporter |
| FTT0487 | major facilitator superfamily (MSF) transporter |
| FTT0496 | hypothetical protein |
| FTT0524 | hypothetical protein |
| FTT0525 | hypothetical protein |
| FTT0528 | ABC transporter |
| FTT0566 | hypothetical protein |
| FTT0572 | proton-dependent oligopeptide transport (POT) |
| FTT0584 | hypothetical protein |
| FTT0602c | hypothetical protein |
| FTT0604 | The Monovalent Cation:Proton Antiporter-1 (CPA1) |
| FTT0623 | trigger factor (TF) protein tig |
| FTT0633 | membrane protease subunit hflK |
| FTT0666c | methylpurine-DNA glycosylase family protein |
| FTT0677c | hypothetical protein |
| FTT0685c | ABC transporter, Voltage-Gated Ion Channel (VIC) |
| FTT0727 | ABC transporter (Exporters:DRI/YHIH) |
| FTT0728 | ABC transporter, ATP-binding protein ybhF |
| FTT0728 | ABC transporter, ATP-binding protein ybhF |
| FTT0729 | ABC transporter, membrane protein |
| FTT0729 | ABC transporter, membrane protein |
| FTT0741c | hypothetical protein |
| FTT0742 | hypothetical lipoprotein |
| FTT0754c | hypothetical membrane protein |
| FTT0775c | major facilitator superfamily (MFS) transport protein |
| FTT0775c | major facilitator superfamily (MFS) transporter bcr2 |
| FTT0829c | aspartate:alanine antiporter |
| FTT0832 | FKBP-type 16 kDa peptidyl-prolyl cis-transisomerase |
| FTT0843 | tRNA-methylthiotransferase MiaB |
| FTT0851 | hypothetical protein |
| FTT0852 | hypothetical protein |
| FTT0865 | pseudogene, hypothetical protein |
| FTT0889c | Type IV pili fiber building block protein |
| FTT0890c | Type IV pili fiber building block protein |
| FTT0903 | hypothetical protein |
| FTT0910 | hypothetical protein |
| FTT0911 | hypothetical protein |
| FTT0932 | ROK family protein |
| FTT0933 | pseudogene, bifunctional protein birA |
| FTT0939c | adenosine deaminase add |
| FTT0947c | major facilitator superfamily (MFS) transporter |
| FTT0961 | mdaB, modulator of drug activity B |
| FTT0975 | hypothetical protein |
| FTT1006 | hypothetical membrane protein |
| FTT1047c | hypothetical protein |
| FTT1048c | hypothetical protein |
| FTT1068c | hypothetical protein (A.I subspecies specific) |
| FTT1071c | hypothetical protein |
| FTT1073c | hypothetical protein |
| FTT1076 | transcription regulator hipA |
| FTT1080c | hypothetical membrane protein |
| FTT1090 | hypothetical membrane protein |
| FTT1091 | isochorismatase hydrolase family protein |
| FTT1106 | tryptophan-rich sensory protein tspO |
| FTT1118c | hypothetical membrane protein |
| FTT1122c | hypothetical lipoprotein |
| FTT1144 | pseudogene, short-chain dehydrogenase/reductase |
| FTT1144 | short-chain dehydrogenase/reductase (SDR) family |
| FTT1172c | cold shock protein (DNA-binding) csp |
| FTT1172c | cold shock protein csp |
| FTT1175c | hypothetical membrane protein |
| FTT1187 | hypothetical protein |
| FTT1188 | hypothetical membrane protein |
| FTT1277c | sodium-solute symporter |
| FTT1286 | pseudogene, choloylglycine hydrolase family protein |
| FTT1307c | hypothetical protein |
| FTT1308c | hypothetical protein |
| FTT1348 | hypothetical protein |
| FTT1360c | hypothetical protein pdpD |
| FTT1361c | hypothetical protein |
| FTT1376 | acyl carrier protein acpP |
| FTT1385c | hypothetical protein |
| FTT1430c | pseudogene, methyltransferase |
| FTT1453c | O-antigen flippase wzx |
| FTT1546 | hypothetical protein |
| FTT1559c | pyrroline-5-carboxylate reductase proC |
| FTT1580c | hypothetical protein |
| FTT1581c | endonuclease |
| FTT1594 | transcriptional regulator lysR family |
| FTT1597 | hypothetical protein |
| FTT1598 | hypothetical membrane protein |
| FTT1614c | hypothetical protein |
| FTT1658c | hypothetical protein |
| FTT1659 | hypothetical protein |
| FTT1661 | thiopurine S-methyltransferase tmpT |
| FTT1703 | hypothetical protein |
| FTT1716c | hypothetical protein |
| FTT1736c | two component sensor protein kdpD |
| FTT1759c | pseudogene, oxidase-like protein |
| FTT1766 | O-methyltransferase |
| FTT1768c | chitinase |
| FTT1781c | hypothetical protein |
| FTT1784c | hypothetical protein |
| FTT1787c | transporter, lysE family |
| FTT1789 | hypothetical protein |
| FTT1791 | hypothetical protein |
